# Process-Directed Self-Assembly of Copolymer Blends: II. Continuous Tuning of Structure Size

**DOI:** 10.1021/acs.macromol.5c02630

**Published:** 2025-10-24

**Authors:** Jiayu Xie, Marcus Müller

**Affiliations:** Institute for Theoretical Physics, 9375 Georg-August University of Göttingen, 37077 Gottingen, Germany

## Abstract

Combining self-consistent field theory and single-chain-in-mean-field simulations, we study the self-assembled morphology and structure size of binary diblock copolymer blends in equilibrium and after processing—such as quenching, annealing, evaporation-induced self-assembly (EISA), and nonsolvent-induced phase separation (NIPS). The equilibrium phase diagrams reveal that adding long linear A_2_B_2_ copolymers to a melt of shorter, cylinder-forming linear A_1_B_1_ copolymers can enlarge the equilibrium cylinder radius by at least 3-fold before macrophase separation sets in. Our particle-based simulations uncover a strong dependence of structure size on processing pathways. Notably, blending A_2_B_2_ copolymers results in a higher magnification of cylinder radii in EISA compared to quenching or annealing. We further analyze how this blending strategy tailors the pore size and transforms the morphological characteristics of integral asymmetric isoporous membranes fabricated by NIPS. By blending in a second diblock copolymer that is 2.25 times the length of the host, a pore-size magnification of up to 70% can be achieved. With further optimization, a more than 2-fold increase appears attainable without significantly compromising membrane quality. Overall, our study offers insights into the structure–processing–property relationships in block copolymer systems and provides design principles for tailoring nanostructures through blend composition and processing strategies across a range of applications.

## Graphical abstract

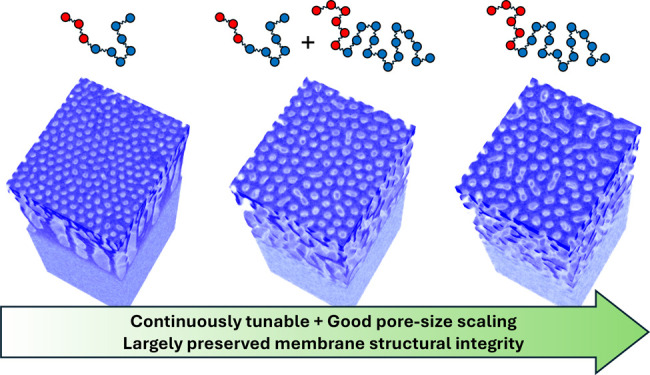

## Introduction

Block copolymers are macromolecules composed of two or more chemically distinct polymer segments, forming blocks that are covalently linked. The chemical incompatibility between these blocks drives phase separation; however, the covalent linkage prevents demixing at macroscopic scales. As a result, block copolymers undergo microphase separation and form a rich variety of self-assembled, spatially modulated morphologies with long-range order. This ability to generate well-defined, tunable nanostructures makes them highly attractive for a wide range of advanced applications, such as drug delivery,^1,2^ photonic crystals,^3,4^ nanoscale lithography,^5–7^ and porous membrane fabrication.^8–10^

The length scale of the self-assembled structures in block copolymers is critical for many practical applications. For example, in photonic materials, the optical properties are directly determined by the periodicity of the nanostructure, which must be on the order of the wavelength of visible or infrared light to effectively manipulate light propagation.^11,12^ Similarly, in block copolymer lithography, the feature size and pattern fidelity are tied to the intrinsic periodic length of the self-assembled morphology.^13,14^ For integral-asymmetric isoporous membranes, the pore size dictates both selectivity and permeability;^10,15,16^ therefore, the pore size must strike a balance: small enough to exclude target solutes, yet large enough to maintain adequate water permeability.

Integral-asymmetric isoporous membranes can be fabricated by a bottom-up two-step process, self-assembly by nonsolvent-induced phase separation (SNIPS), which combines evaporation-induced self-assembly (EISA) with nonsolvent-induced phase separation (NIPS). In SNIPS, a dilute, homogeneous solution of a cylinder-forming copolymer and typically two solvents (one volatile, one nonvolatile) is prepared. During EISA, evaporation of the volatile solvent increases the copolymer concentration near the liquid–gas interface (skin formation), driving self-assembly into cylinders oriented perpendicular to the membrane surface. Once a sufficiently thick microphase-separated top layer has formed, NIPS is triggered by immersion into a nonsolvent bath. Rapid solvent–nonsolvent exchange through the cylindrical channels then opens pores in the top layer, and macrophase separation between the polymer and nonsolvent generates a macroporous substructure that mechanically supports the membrane. The average pore size of the fabricated membrane is ultimately determined by the cylinder dimensions established during copolymer self-assembly.

While the spacing and size of the domain self-assembled by block copolymers can be adjusted by varying the copolymer’s degree of polymerization, this approach requires synthesizing a new copolymer for each desired length scale and optimizing the nonequilibrium fabrication process. Developing cost-effective techniques to achieve tunable structure sizes could broaden the utility of block copolymers across diverse applications. A more economical yet effective strategy for controlling structure size is to use blends containing block copolymers. By adjusting the blend composition, one can continuously tune the domain size and periodicity of self-assembled structures without the need for customized polymer synthesis. The incorporation of selective homopolymers is a well-established strategy to swell specific microdomains in block copolymer systems,^17–29^ thereby increasing the domain size and periodicity of the resulting ordered morphology. However, the utility of homopolymers as domain-size tuners is often constrained by their limited miscibility with the host copolymers.^19,20,25,27,30–32^ Blending homologous block copolymers with different molecular weights, block compositions and/or architectures offers an alternative, more robust approach. These copolymers can act as cosurfactants, with their junction points localizing at domain interfaces to reduce interfacial tension.^33,34^ This promotes the coassembly of different copolymers into well-ordered microdomains while suppressing macrophase separation. A straightforward blending strategy involves binary mixtures of two linear diblock copolymers, A_1_B_1_ and A_2_B_2_, which differ in chain length and/or block composition. Previous experiments have demonstrated that such bidisperse diblock copolymer blends can effectively modulate the pore size in integral-asymmetric isoporous membranes.^35^

While block copolymer blends hold great promise for tailoring self-assembled structure sizes in a wide range of applications, key aspects of their phase behavior remain poorly understood, even in the relatively simple case of bidisperse diblock copolymers. Despite the cosurfactant effect, a notable composition window exists where macrophase separation occurs when the chain-length disparity between the two copolymers is large.^36–39^ Such demixing will impede the formation of structures with a uniform length scale throughout the sample. More importantly, differences in chain length introduce significant disparities in the dynamics and degree of segregation between the constituent copolymers, which can affect the final self-assembled morphology, depending on the specific processing pathway. In particular, it remains an open question whether, and by what mechanisms, such systems attain equilibrium under different processing pathways. These factors may critically influence the ability of diblock copolymer blends to achieve target feature sizes.

In a previous work (Paper I),^39^ we demonstrated that different processing conditions, coupled with micro- and macrophase separation, can lead to qualitatively distinct morphologies in binary diblock copolymer blends. Complementing that study, the present work quantitatively investigates how the structure size scales with the proportion of the longer copolymer in the blends under different processing conditions. To this end, we employ self-consistent field-theory (SCFT) and particle-based simulations to compare the equilibrium phase behavior and nonequilibrium self-assembly of binary diblock copolymer blends. Focusing on cylinder-forming systems, we first construct the equilibrium phase diagrams and evaluate the optimal cylinder size and periodicity using SCFT. Subsequently, we investigate the dynamical structure formation from the disordered phase to ordered morphologies for various processing pathways, including thermal quenching, gradual cooling, and solvent evaporation. To capture both the collective density evolution and the single-chain dynamics, we employ particle-based simulations of a soft, coarse-grained model in conjunction with the single-chain-in-mean-field (SCMF) algorithm.^40–42^ We provide a detailed analysis of how introducing a second, longer copolymer influences the resulting structures and increases the characteristic length scale of the system. As a specific application, we systematically examine how blending in longer chains can be used to tune the pore size in integral-asymmetric, isoporous membranes fabricated via evaporation-induced self-assembly (EISA) and nonsolvent-induced phase separation (NIPS).^8–10,43,44^ We further explore the influence of key system parameters on the final membrane morphology and estimate the range of pore sizes achievable through this strategy. Our results provide a comprehensive understanding of the self-assembly of diblock copolymers under different ordering pathways and offer valuable insights into designing materials with tailored feature sizes for a variety of applications.

## Theory and Simulation Methods

To determine the equilibrium morphology and the optimal periodicity of bidisperse blends, we employ SCFT.^45–51^ Subsequently, we utilize SCMF simulations of a soft, highly coarse-grained particle-based model to investigate the kinetics of structure formation and explore the resultant structure sizes of nonequilibrium metastable states. To guide the selection of simulation parameters for viable membrane casting solutions, we employ the random-phase approximation (RPA)^27,45,52,53^ to analyze the spinodal instability of the system. A common molecular representation – the discrete Gaussian chain model – is used across all techniques in this study. Here, we provide a brief description of the molecular model and introduce the relevant parameters; detailed aspects of the theoretical and simulation frameworks are deferred to the Supporting Information (SI).

The most complex system analyzed by SCFT and RPA corresponds to the casting solution utilized for fabricating porous membranes via EISA and NIPS. This casting solution consists of two species of diblock copolymers, A_1_B_1_ and A_2_B_2_, mixed with a volatile solvent, S, and a nonvolatile solvent, C, forming a quaternary mixture. The SCFT and RPA are formulated for this quaternary system, which can be readily reduced to a binary blend of diblock copolymers by excluding the solvents. Details of the parameter definitions, thermodynamic and statistical-mechanical formulations, as well as the numerical procedures of SCFT and RPA, are provided in section Self-Consistent Field Theory (SCFT) and section Random-Phase Approximation (RPA), respectively, of the SI. Throughout this work, we fix the number of segments in the A_1_B_1_ chain at *N*_1_ = 64 and use it as the reference chain length. The key model parameters are the A-block compositions of the two copolymers (*f*_1_ and *f*_2_), the chain-length ratio between the long A_2_B_2_ copolymer and the short A_1_B_1_ copolymer (γ_2_ = *N*_2_/*N*_1_), the set of χ_αβ_ (α, β ∈ {A, B, S, C} with β ≠ α) parameters quantifying the interaction strengths between different segment types, and the average concentrations of different molecular species in the system (ϕ̅_1_, ϕ̅_2_, ϕ̅_S_ and ϕ̅_C_). Given a set of candidate phases, we can employ SCFT to obtain their thermodynamic potentials and to construct equilibrium phase diagrams on phase planes spanned by system parameters of interest. Unless otherwise specified, we include lamellae (LAM), double gyroid (DG), hexagonally packed cylinders (HEX), body-centered cubic (BCC) spheres, as well as the disordered (DIS) phase, as the default set of candidate phases in our calculations.

With the thermodynamic equilibrium reference obtained from SCFT, the dynamical and nonequilibrium behavior of the polymer blends is investigated using Monte Carlo (MC) simulations accelerated by the SCMF algorithm.^41,54^ To investigate the structure formation during SNIPS by SCMF simulations, we incorporate one additional molecular species, i.e., either a gas G (during EISA) or a nonsolvent N (during NIPS). In our simulations, we bias the local MC trial displacements of the beads using the strong bonded forces,^55^ resulting in Rouse-like dynamics.^56^ The time unit, τ_*R*_, is set by the time required for a linear, noninteracting polymer with *N*_1_ segments to diffuse its end-to-end distance (*R*_*e*_). For binary diblock copolymer blends processed by quenching or annealing, the simulations are conducted in a quasi-two-dimensional (2D) cell of dimensions 24 × 21 × 1*R*_*e*_^3^, whereas a much larger cell of 24 × 21 × 50*R*_*e*_^3^ is employed for the SNIPS simulations. A detailed introduction of the simulation framework, including the simulation protocol, simulation-cell setup, and techniques used to capture nonequilibrium phenomena,^10,40,43,44,57^ is given in section Particle-Based Monte Carlo Simulation of the SI. All simulations are performed using the open-source software SOft coarse grained MC Acceleration (SOMA).^42^

## Results and Discussion

### Equilibrium Phases in Binary A_1_B_1_/A_2_B_2_ Blends

We begin our investigation by constructing equilibrium phase diagrams for binary blends of A_1_B_1_ and A_2_B_2_ diblock copolymers using SCFT. The equilibrium phase diagrams have been thoroughly mapped out in prior studies.^36–38,58,59^ Thus, we focus on case-specific parameter sets that serve as reference points for our subsequent simulations. We focus on the cylindrical HEX phase that is relevant for fabricating isoporous block copolymer membranes and examine how blending a second diblock copolymer into a cylinder-forming diblock copolymer affects the equilibrium morphology and characteristic length scale. Specifically, the SCFT phase diagrams help identify the parameter space in which the domain size and spacing of the HEX phase remain continuously tunable over a wide range in equilibrium, without interruption by macrophase separation or competing morphologies. This establishes the equilibrium thermodynamics for investigating the cylinder- and pore-size regulation via the incorporation of a second long copolymer into a short-copolymer melt and SNIPS-fabricated membranes, which we will considered in our particle-based simulations (*vide infra*).

We consider the case where A_1_B_1_ is a short diblock (*N*_1_ = 64) that forms A-rich cylinders, and A_2_B_2_ is a longer diblock (*N*_2_ = γ_2_ *N*_1_ > *N*_1_) with an A-block composition that is equal to or greater than that of A_1_B_1_ (*f*_2_ ≥ *f*_1_). In Figure 1a, we present the ϕ̅_2_–γ_2_ phase diagram, constructed using SCFT with *f*_1_ = *f*_2_ = 5/16 and χ_AB_ *N*_1_ = 30. The HEX morphology remains the stable phase throughout the entire composition range for 1 ≤ γ_2_ ≤ 1.75. For larger chain-length asymmetry, γ_2_ ≥ 2, the DG phase appears as the stable phase within a narrow composition window close to ϕ̅_2_ = 1. Figure 1b extends the exploration to higher values of γ_2_, ranging from 4 to 6. At these values, the disparity in segregation strength between A_1_B_1_ and A_2_B_2_ copolymers becomes significant, making the convergence of the SCFT equations challenging. For instance, at χ_AB_ *N*_1_ = 30 and γ_2_ = 6, the effective χ_AB_ *N*_2_ = γ_2_χ_AB_ *N*_1_ for A_2_B_2_ reaches 180. To facilitate convergence in such cases, we reduce the incompatibility to χ_AB_ *N*_1_ = 17.5. For the same reason, it becomes difficult to obtain solutions for the DG phase when γ_2_χ_AB_ *N*_1_ > 100. Nevertheless, extrapolating from the trend at lower γ_2_, we expect the stability window of the DG phase to remain narrow close to ϕ̅_2_ = 1 even at higher γ_2_, and thus it is omitted for γ_2_χ_AB_ *N*_1_ > 100. Notably, although the HEX phase still appears to be the stable phase over almost the entire composition range, even at γ_2_ = 4, a stability window for the BCC phase emerges at γ_2_ ≳ 5 around ϕ̅_2_ ≈ 0.45, with a width Δϕ̅_2_ ≈ 0.16 at γ_2_ = 5. This BCC region splits the HEX stability window into two separate branches along the composition axis. Between the BCC phase and each adjacent HEX branch lies a two-phase coexistence region, denoted 2ϕ. In these phase coexistence regions, the thermodynamic equilibrium state of the blend involves macrophase separation, with each phase enriched (relative to the other coexisting phase) in one of the copolymer species. The phase coexistence region to the left of the BCC window is noticeably wider than the one on the right. At a higher value of γ_2_ = 6, the topology of the phase diagram remains identical to that at γ_2_ = 5. The phase coexistence and BCC regions expand further at the expense of both the left and right HEX stability windows. In particular, the phase coexistence region to the left of the BCC phase expands substantially compared with that observed at γ_2_ = 5. The BCC phase on the right side of this phase coexistence region (the “large” phase) exhibits a significantly larger characteristic length scale than the HEX phase on the left (the “small” phases), due to their much higher content of the longer A_2_B_2_ copolymers. The emergence and expansion of the phase coexistence region with increasing γ_2_ indicate that, when the microdomains self-assembled from the two individual copolymers differ greatly in size, incorporating them into a common microstructure becomes highly frustrated. As discussed in Paper I,^39^ this thermodynamically favors macrophase separation despite the associated loss in translational entropy.

**Figure 1:**
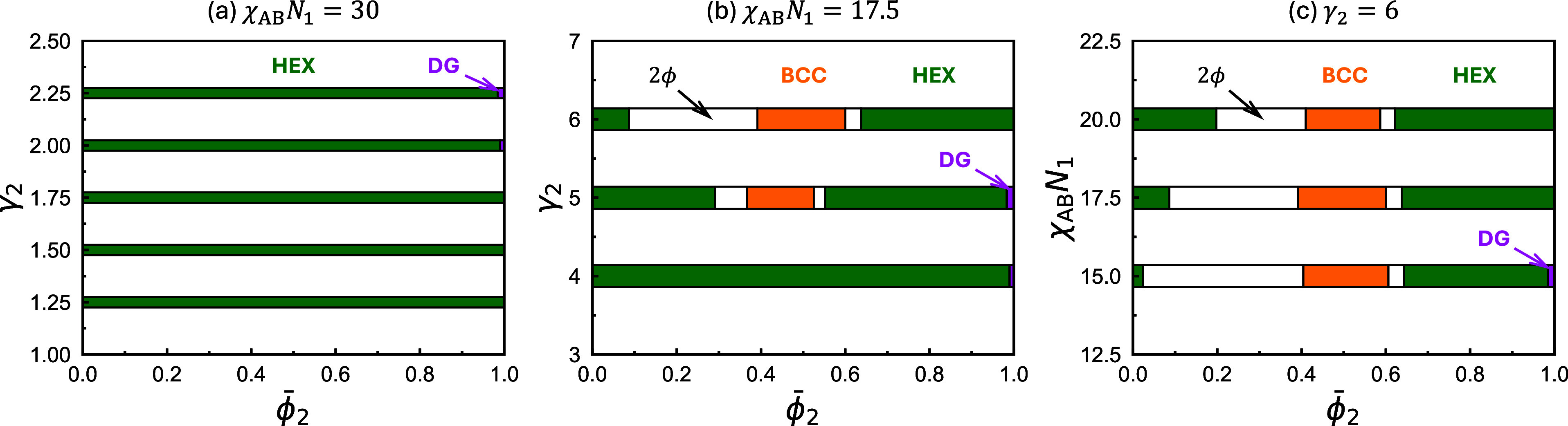
Equilibrium phase diagrams constructed using SCFT for binary blends of diblock copolymers with identical A-block fraction *f*_1_ = *f*_2_ = 5/16, but different chain-length ratios γ_2_ = *N*_2_/*N*_1_ or incompatibilities χ_AB_ *N*_1_. The relevant parameters used to construct each diagram are indicated above their respective plots.

Figure 1c presents a ϕ̅_2_–χ_AB_ *N*_1_ phase diagram to assess the influence of the incompatibility χ_AB_ *N*_1_ at fixed γ_2_ = 6. As χ_AB_ *N*_1_ increases from 15 to 17.5 and then to 20, the stability window of the BCC phase and the right branch of the HEX phase, along with their corresponding phase coexistence region, remain largely unchanged. In contrast, the wide phase coexistence region between the left HEX branch and the BCC phase contracts, allowing the stability window of the left HEX branch to expand. The reduced tendency for macrophase separation with increasing χ_AB_ *N*_1_ stems from an enhanced segregation of the long B blocks of the A_2_B_2_ chains at the interstitial regions, which alleviates the packing frustration, thereby promoting the coassembly of the two copolymers into the same microstructure and mitigating macrophase separation.^39^

Figure 2 presents four additional phase diagrams, exploring blends that contain the same A_1_B_1_ copolymers as in Figure 1, but with A_2_B_2_ copolymers characterized by varying *f*_2_ values greater than *f*_1_. In all cases shown in Figure 2a–d, the stable phase for the neat A_2_B_2_ copolymers, ϕ̅_2_ = 1, is the LAM phase. Consequently, as ϕ̅_2_ increases, the phase progression HEX → DG → LAM is generally observed. Between the stability regions of any two adjacent phases along the blend-composition axis, a phase coexistence window appears; however, their widths are relatively narrow in Figure 2a–c. For all *f*_2_ values, the stability window for the DG phase decreases as γ_2_ increases. Although the DG window fully closes only in Figure 2b, the observed trend suggests that it will eventually vanish in all cases for larger γ_2_. Moreover, as γ_2_ increases, all phase boundaries systematically shift toward larger ϕ̅_2_, leading to an expansion of the HEX region at the expense of the DG and LAM phases. Figure 2d explores large γ_2_ values at *f*_2_ = 0.5 with a reduced χ_AB_ *N*_1_ = 20. Similar to Figure 1b, a wide phase coexistence region emerges, separating the ordered one-phase regions into two distinct, small and large, branches. However, in contrast to Figure 1b,c, the wide phase coexistence region is flanked by the small and large HEX branches, without the appearance of the large BCC phase. Despite differences in the polymer-chain model and parameter choices, the overall phase behavior depicted in Figures 1 and 2 is qualitatively consistent with previous theoretical studies.^37,38^

**Figure 2:**
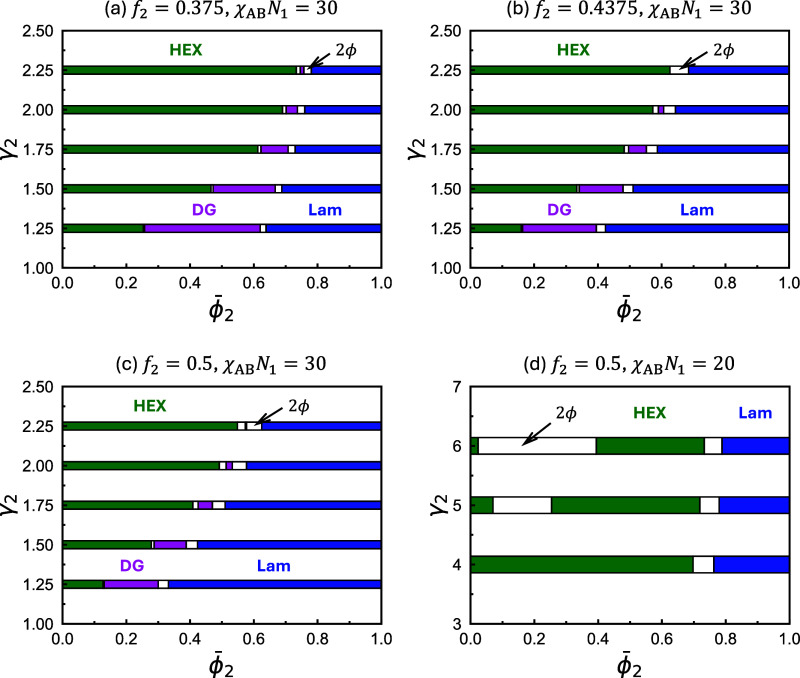
ϕ̅_2_-γ_2_ phase diagrams constructed using SCFT for binary blends of diblock copolymers with fixed *f*_1_ = 5/16 for the A_1_B_1_ copolymer, different incompatibilities χ_AB_ *N*_1_, and different *f*_2_ for the A_2_B_2_ copolymer. The relevant parameters used to construct each diagram are indicated above their respective plots.

### Equilibrium Domain Sizes in Binary A_1_B_1_/A_2_B_2_ Blends

We now turn our attention to the optimal characteristic length scale in the HEX morphology of the blends, as summarized in Figures 3 and 4. The parameters used in both figures correspond one-to-one to those employed in Figures 1 and 2, respectively. Two quantities are evaluated: the intercolumnar distance, *d*_c_ (solid curves), and the radius of the A-rich core, *r*_c_ (dashed curves). To assess how the length scale is controlled by incorporating A_2_B_2_ copolymers into the morphology of A_1_B_1_ copolymers, all curves are normalized by their values at ϕ̅_2_ = 0, yielding the magnification ratios. For *f*_1_ = *f*_2_ = 5/16 and relatively small γ_2_ (Figure 3a), *d*_c_ and *r*_c_ closely follow each other and increase approximately linearly with ϕ̅_2_. At very large γ_2_ (Figure 3b), *r*_c_ grows moderately faster than *d*_c_ as ϕ̅_2_ increases. More importantly, at large γ_2_, the nonlinearity becomes pronounced. Specifically, for small ϕ̅_2_, all curves grow slowly with ϕ̅_2_, but the magnification ratio increases relatively rapidly within the range 0.3 ≲ ϕ̅_2_ ≲ 0.5, before transitioning to a roughly linear, rapid-growth regime. This trend is consistent with previous theoretical results for the lamellar phase.^36^ Figure 3c further examines the influence of χ_AB_ *N*_1_, showing that a small increase in χ_AB_ *N*_1_ slightly enhances the nonlinearity.

**Figure 3:**
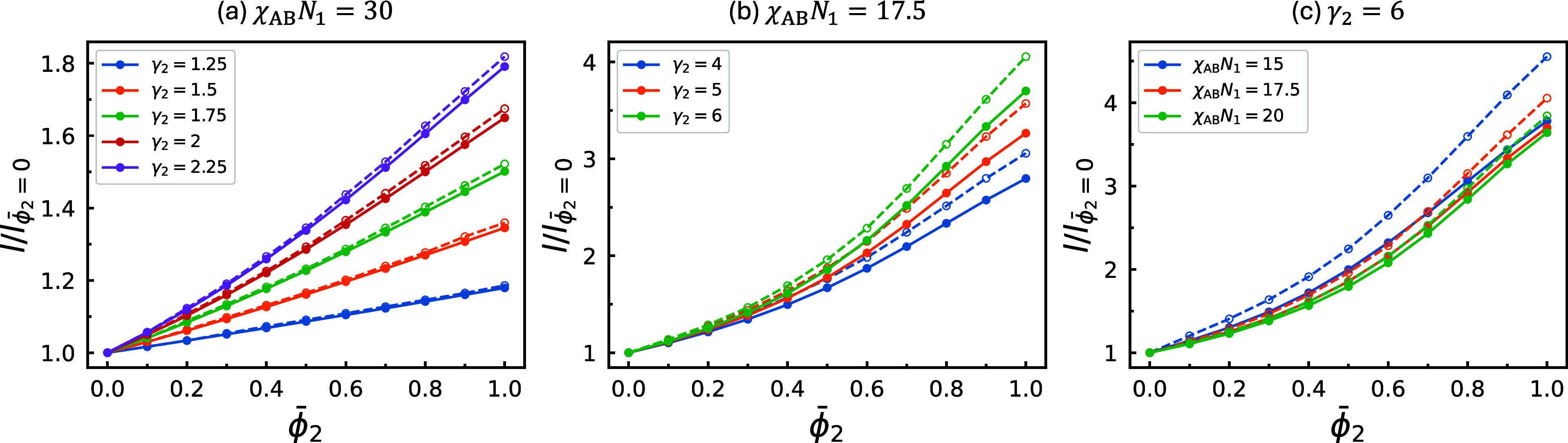
Equilibrium intercolumnar distance, *d*_c_ (solid curves), and radius of the A-rich core, *r*_c_, evaluated along the blend-composition paths in the corresponding phase diagrams in Figure 1. All data points along each curve are normalized to the value at ϕ̅_2_ = 0, yielding the magnification ratio of the corresponding length scale as a function of ϕ̅_2_.

**Figure 4:**
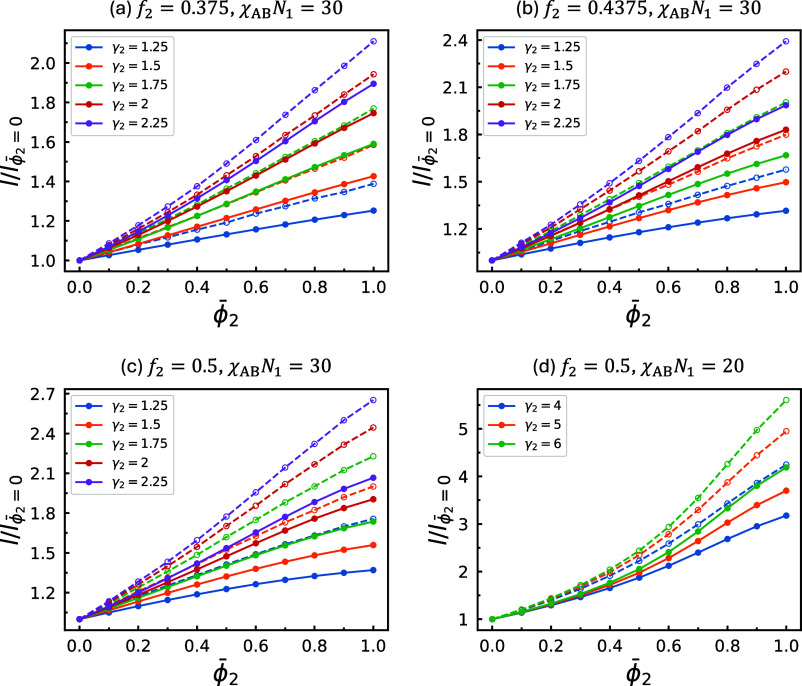
Equilibrium intercolumnar distance, *d*_c_ (solid curves), and radius of the A-rich core, *r*_c_ (dashed curves), evaluated along the blend-composition paths in the corresponding phase diagrams in Figure 2. All data points along each curve are normalized to the value at ϕ̅_2_ = 0, yielding the magnification ratio of the corresponding length scale as a function of ϕ̅_2_.

Figure 4 shows the magnification ratios of *d*_c_ and *r*_c_ in cases where *f*_2_ > *f*_1_. The overall trends observed in Figure 4 generally mirror those in Figure 3, with two major distinctions: First, when *f*_2_ > *f*_1_, increasing the concentration of A_2_B_2_ copolymers raises the total A-block fraction, which in turn leads to a larger growth rate in *r*_c_ compared to *d*_c_. This behavior is particularly useful for creating larger A-rich domains that are packed more densely together, and it has already been effectively utilized to stabilize complex structures in sphere-forming systems.^34,58–62^ Second, when *f*_2_ is large and γ_2_ is small, the rate of increase in magnification ratio decreases with ϕ̅_2_. This effect is most evident for *d*_c_ at *f*_2_ = 0.5 and γ_2_ = 1.25, where the curve noticeably flattens as it approaches high ϕ̅_2_ values, showing a clear contrast to the corresponding curve in Figure 3a. We also found that these flattening trends in *d*_c_ and *r*_c_ correlate with the depletion of the majority B_2_ blocks of the long copolymers from the interstitial voids. This is demonstrated in Figure S1, where the B_2_ distributions at *f*_2_ = 0.3125 and 0.5 are compared at γ_2_ = 1.25 for different values of ϕ̅_2_. The observed depletion is driven by the preferential localization of the more symmetric A_2_B_2_ copolymers at the portions of internal interfaces with lower curvature, located near the faces of the Wigner–Seitz cells. At small γ_2_, this curvature-driven localization outweighs the tendency of the long chains to stretch into the interstitial voids, which correspond to the vertices of the Wigner–Seitz cells. However, the competition reverses when γ_2_ becomes large, as shown in Figure S2, where a similar comparison is made at γ_2_ = 2.25. At this higher γ_2_ value, the B_2_ blocks consistently segregate to the interstitial voids regardless of *f*_2_. Finally, in Figure 4d, strong nonlinearity appears at very large γ_2_, consistent with the observations in Figure 3b,c.

Although Figures 3 and 4 show that the equilibrium characteristic length scale of the HEX phase – measured by either *d*_c_ or *r*_c_ – can be effectively tuned in a continuous manner in binary blends by adjusting the blend composition, not all values are accessible according to the equilibrium phase diagrams. Theoretically, the applicable range of γ_2_ is limited by the macrophase separation between small and large phases. This macrophase separation window occurs at high values of γ_2_, regardless of the value of *f*_2_, and expands upon further increasing γ_2_. Furthermore, although the addition of lamella-forming A_2_B_2_ copolymers can more effectively enlarge *d*_c_, it can also give rise to order–order phase transitions, destroying the desired HEX morphology. Nonetheless, within the parameter space explored here, the equilibrium length scale of the HEX phase can be continuously enlarged by about a factor of 3 (for *f*_2_ = 0.3125 and γ_2_ = 4). With further parameter optimization, the magnification ratio in the equilibrium length scale accessible by adding the longer diblock chains can be expected to become even larger.

### Process-Dependent Magnification Ratios in Cylinder Size: Quench, Annealing, and EISA

The SCFT-predicted phase diagrams and the length scales in the HEX morphology shown in Figures 1–4 provide a clear picture of the equilibrium behavior of the binary A_1_B_1_/A_2_B_2_ blends. A large, continuous range is identified over which the equilibrium cylinder size and lattice spacing can be tuned. However, the blends feature a complex free-energy landscape with a multitude of local minima characterized by different length scales. As a result, the system could be easily trapped in different metastable states depending on the specific processing conditions employed to induce ordering.^39^ Therefore, it is unclear if, and how, the system can approach the equilibrium morphology and length scale predicted by SCFT. To understand this question, in this section, we use particle-based simulations to explore the dynamical process of structure formation under different processing conditions, such as quenching, annealing and EISA.

As suggested by the equilibrium SCFT calculations, although using A_2_B_2_ copolymers with *f*_2_ > *f*_1_ = 5/16 leads to a more rapid increase in cylinder size with increasing ϕ̅_2_, the reduced stability window of the HEX phase, caused by order–order phase transitions, ultimately results in a comparable maximal magnification ratio as in the case of *f*_1_ = *f*_2_ = 5/16. For simplicity, we restrict our investigation by SCMF simulations to the latter case (*f*_1_ = *f*_2_). To this end, we conduct simulations at γ_2_ = 1.5 and 2.25. These choices of γ_2_ lie well below the regime where the BCC phase and a wide phase coexistence region emerge (c.f. Figure 1a), and are expected to result exclusively in microphase separation regardless of processing conditions.

#### Quench and Annealing

We now investigate how increasing the concentration of A_2_B_2_ copolymers enhances the characteristic length scale of the cylindrical morphology under two typical processing protocols, i.e., sudden quench and thermal annealing. In both protocols, the simulations begin at *t* = 0 with the disordered state where all polymer chains are homogeneously distributed in the simulation cell, corresponding to χ_AB_ *N*_1_ = 0. Ordering is then induced either by an abrupt quench or by gradual annealing to a target χ_AB_ *N*_1_, as illustrated in Figure 5. During annealing, χ_AB_ *N*_1_ increases linearly and remains at the target value once reached.

**Figure 5:**
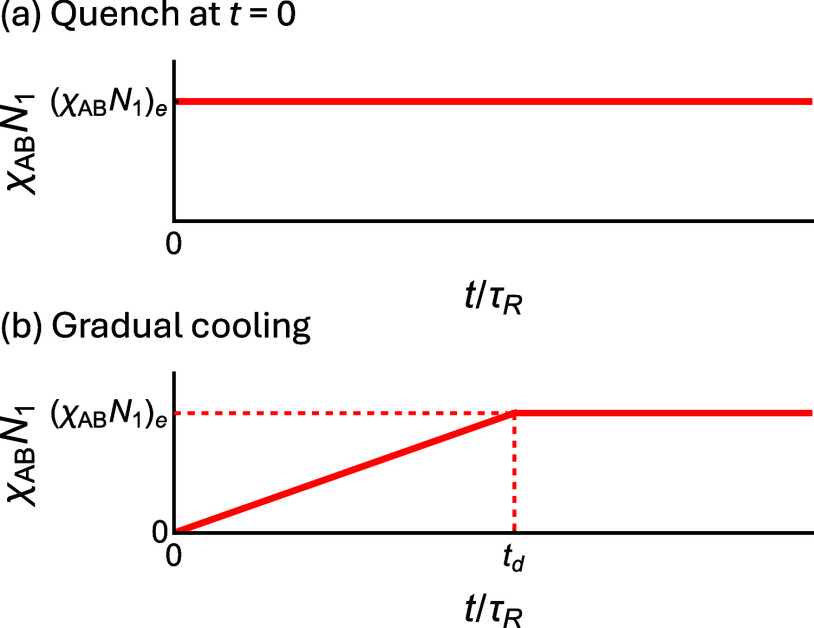
Time evolution of χ_AB_ *N*_1_ in the (a) quenching and (b) gradual cooling (annealing) processes. In the quenching process, χ_AB_ *N*_1_ is instantaneously increased from 0 to the target value (χ_AB_ *N*_1_)_e_ at *t* = 0. During the annealing process, χ_AB_ *N*_1_ increases linearly from 0 at *t* = 0 to (χ_AB_ *N*_1_)_e_ at *t* = *t*_d_, after which it remains constant.

For both the quenching and annealing processes, simulations are carried out at γ_2_ = 1.5 and 2.25; at each γ_2_, ϕ̅_2_ values from 0 to 1 in increments of 0.25 are examined. Starting from the DIS phase (corresponding to χ_AB_ *N*_1_ = 0), all simulations target the final thermodynamic state points at χ_AB_ *N*_1_ = 30. All other parameters are identical to those in Figure 1a. For the annealing process toward each final state point, three annealing durations are considered: 3τ_*R*_, 6τ_*R*_, and 12τ_R_. All quenching and annealing simulations are run for a total time of 12τ_*R*_.

As an example, Figure 6 shows the time evolution of the total A-block density for a quenching simulation (top panel) and an annealing simulation with an annealing duration of 12τ_*R*_ (bottom panel), both at γ_2_ = 2.25 and ϕ̅_2_ = 0.5. In the quenching simulation, phase separation sets in rapidly after *t* > 0. By *t* = 0.2τ_*R*_, the A-rich component has already organized into predominantly discrete, circular domains with only faint remnants of finger-like connectivity. At *t* = 2τ_*R*_, these domains are fully separated due to the overall asymmetric block composition, resulting in a dispersed droplet morphology. The subsequent evolution involves only slow coarsening and interfacial relaxation, yielding a stable array of well-defined A-rich cylinders by *t* = 12τ_*R*_. In contrast, domain formation during annealing proceeds much more gradually. At *t* = 4τ_*R*_, χ_AB_ *N*_1_ has only slightly surpassed the spinodal value of the blend, leading to weak concentration fluctuations. By *t* = 5τ_*R*_, distinct A-rich domains begin to emerge, which subsequently grow and arrange more regularly. At *t* = 7τ_*R*_, most domains have already adopted a nearly circular shape, and by *t* = 12τ_*R*_ the system reaches a well-ordered, strongly segregated cylindrical morphology. The distinct density evolutions during structure formation upon quenching and annealing lead to different morphologies at *t* = 12τ_*R*_. In what follows, we quantitatively analyze the magnification ratios of the average cylinder radius resulting from quenching and annealing when blending different proportions of the second long copolymer.

**Figure 6:**
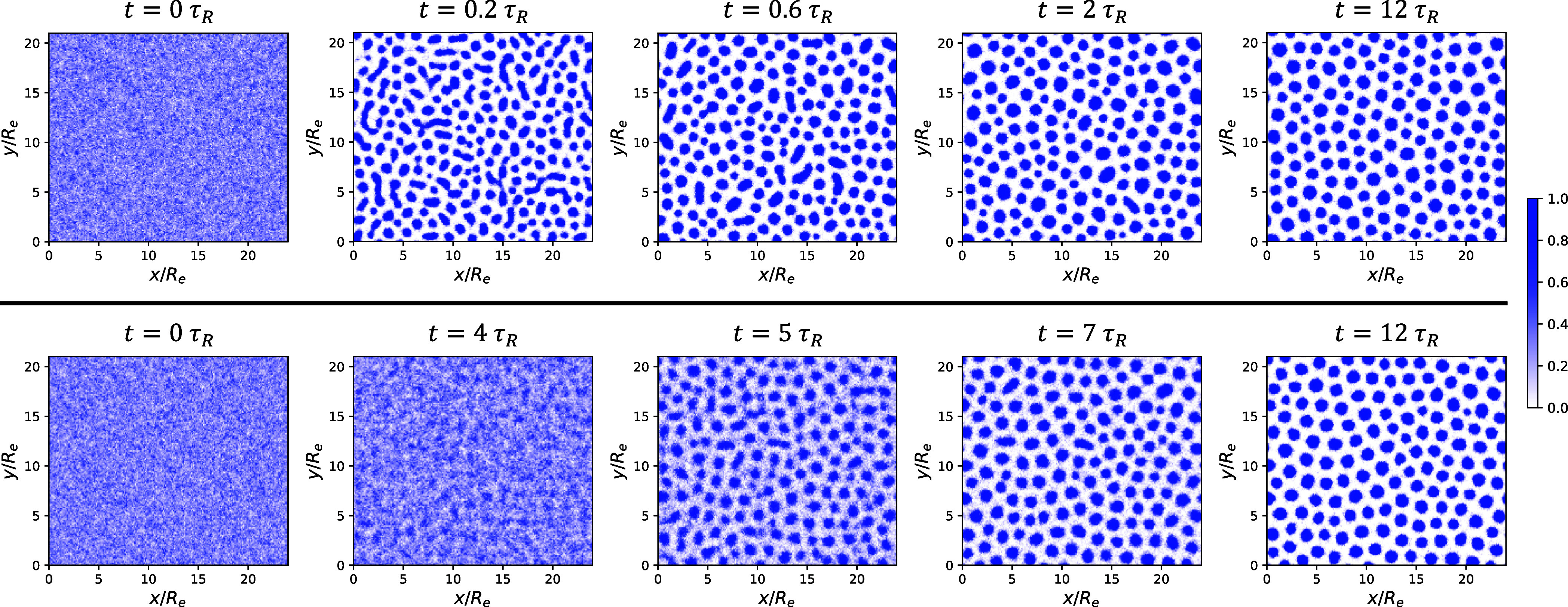
Time evolution of the total A-block density for a quenching simulation (top panel) and an annealing simulation (bottom panel) with a duration of 12τ_*R*_. Both simulations start from the DIS phase corresponding to χ_AB_ *N*_1_ = 0 and target the same final state point at χ_AB_ *N*_1_ = 30, γ_2_ = 2.25, and ϕ̅_2_ = 0.5. In each plot, the displayed density is normalized at every spatial point so that the sum of all components equals unity, yielding composition profiles expressed as local volume fractions. This normalization is applied consistently to all density maps presented in this work, resulting in a consistent color bar ranging from 0 to 1.

Starting with the quenching simulations, we identify individual A-core cylinders and extract their average radii at the final time (*t* = 12τ_*R*_), following the procedure described in section Post-Processing of Density Data from Simulations of the SI. The magnification ratios, computed from the corresponding average radii, are shown in Figure 7 as open red circles. Each data point is computed as the mean value from three independent stochastic realizations of the ordering process, with error bars representing the standard error. For comparison, the SCFT predictions are also plotted as a dashed curve. For both γ = 1.5 and 2.25, the magnification ratios obtained from quenching are consistently lower than those predicted by SCFT, with the deviation increasing as ϕ̅_2_ rises. In the limiting case where all short copolymers are replaced by long ones (ϕ̅_2_ = 1), the percentage increase in cylinder radius achieved via quenching is less than half of the SCFT value for both γ_2_ values, with a slightly larger reduction observed at γ_2_ = 2.25.

**Figure 7:**
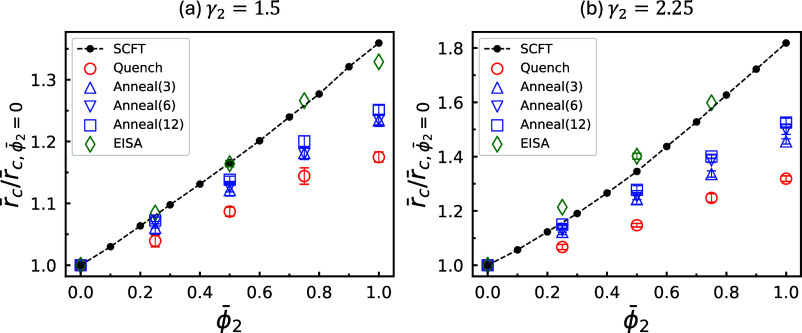
Magnification ratios of the average cylinder radius induced by increasing the proportion, ϕ̅_2_, of the long A_2_B_2_ copolymers for (a) γ_2_ = 1.5 and (b) 2.25. All other parameters are identical to those in Figure 1a. Black solid circles connected by dashed lines denote equilibrium values predicted by SCFT, while the remaining data points are obtained from SCMF simulations under various processing conditions. For EISA data points, the horizontal abscissa corresponds to ϕ̅_2_^bi^.

The resulting magnification ratios from the annealed simulations are shown in Figure 7 as open blue symbols with distinct shapes. These ratios are consistently higher than those obtained from quenching, yet remain systematically below the SCFT predictions. At both γ_2_ values, longer annealing durations lead to higher ratios; however, the variation across the different annealing durations remains minimal.

The reduction in the magnification ratio of the cylinder radius observed in the simulations, relative to the equilibrium SCFT prediction, arises from the increased degree of segregation as more long copolymers are blended into the system. Specifically, with the same χ_AB_, the longer A_2_B_2_ copolymers have a stronger segregation degree, χ_AB_ *N*_2_, due to their higher degree of polymerization, *N*_2_, compared to the shorter A_1_B_1_ copolymers. In addition to a more rugged free-energy landscape, a stronger segregation leads to (i) a smaller fastest-growing length scale during the early stage of spinodal decomposition, but (ii) a larger equilibrium length scale.

The difference between these two length scales upon quenching as a function of the volume fraction of long copolymers is illustrated in Figure 8a, for a specific set of parameters. The fastest-growing length scale is estimated by identifying the maximum of −*k* ^2^ *S* ^–1^(*k*), where *S* ^–1^(*k*) is the inverse collective structure factor obtained by RPA. The equilibrium length scale is represented by the optimal intercolumnar distance predicted by SCFT. For neat A_1_B_1_ copolymers (ϕ̅_2_ = 0), a clear gap between the two length scales is already present, and it widens progressively with increasing ϕ̅_2_. For a system quenched from the disordered phase, as it evolves from the initial spinodal phase separation toward equilibrium, the characteristic length scale must coarsen from that of the fastest-growing mode to the equilibrium one, as indicated by the vertical arrows in Figure 8a. In a strongly segregated HEX phase, this coarsening primarily occurs through many individual fusion events, where smaller cylindrical domains merge to form larger ones, each requiring the system to overcome an associated free-energy barrier.[Fn fn1] Therefore, the system becomes easily trapped in metastable states during coarsening, preventing it from reaching equilibrium. This trapping becomes more long-lived at higher ϕ̅_2_, due to a higher free-energy barrier for cylinder fusion and a larger length-scale gap that must be overcome through successive fusion events. This explains the smaller increase in cylinder-size magnification ratio measured in the quenching simulations compared to the equilibrium SCFT predictions.

**Figure 8:**
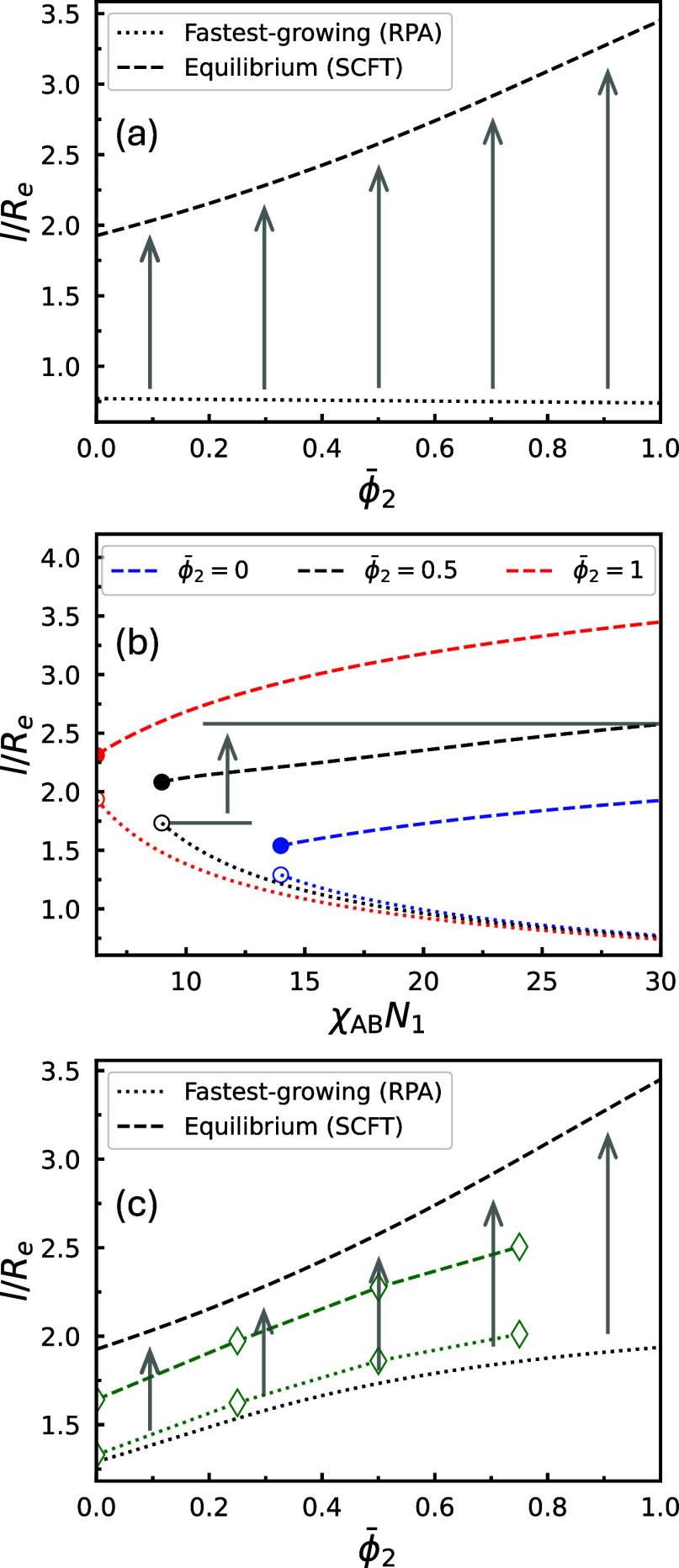
Comparison between the fastest-growing length scale during the early stage of spinodal decomposition from the DIS phase and the equilibrium length scale for various blend compositions and processing conditions: (a) quenching to χ_AB_ *N*_1_ = 30 for blends with varying ϕ̅_2_; (b) quenching to different χ_AB_ *N*_1_ values for selected blend compositions (ϕ̅_2_ = 0, 0.5, and 1); and (c) slow annealing to χ_AB_ *N*_1_ = 30 for blends with varying ϕ̅_2_. In all cases, *f*_1_ = *f*_2_ = 5/16 and γ_2_ = 2.25 are held constant. In (b), line styles (dashed or dotted) follow the convention shown in the legend in (a), regardless of color. For each ϕ̅_2_ in (b), the curves begin at the corresponding spinodal point, indicated by an open circle for the dotted line and a solid circle for the dashed line. Gray arrows in all panels schematize the length-scale paths the blends have to coarsen to reach equilibrium. Green diamonds in (c) are evaluated for the EISA simulations (see, e.g., the bottom row of Figure 13). For the EISA data, the *x* axis represents ϕ̅_2_^bi^.

For a system annealed from the disordered phase, the gap between the two length scales is reduced but still remains. Figure 8b shows the fastest-growing and equilibrium length scales upon quenching as a function of the target χ_AB_ *N*_1_ value for three different blend compositions. All other molecular parameters are identical to those in Figure 8a. During annealing, χ_AB_ *N*_1_ increases gradually over time. In the case of extremely rapid annealing with a duration far shorter than 1τ_*R*_, the system is expected to evolve similarly to a direct quench, as the gap between the two length scales is close to that shown in Figure 8a. This limiting case corresponds to χ_AB_ *N*_1_ = 30 in Figure 8b. In contrast, during much slower annealing, the initial phase separation occurs while the system remains near the spinodal point, resulting in an appreciably larger fastest-growing length scale. In this case, the length-scale gap that the system must coarsen through can be approximated by the difference between the open circle and the final, highest point of the corresponding dashed curve in Figure 8b, as indicated by the gray arrow for the specific case of ϕ̅_2_ = 0.5.

Figure 8c presents the two length scales as a function of ϕ̅_2_ over the entire blend composition range in the limit of slow annealing. Compared to quenching, annealing the system to the same state point yields a larger fastest-growing length scale overall, thereby narrowing the length-scale gap the system must overcome to reach equilibrium.[Fn fn2] Nonetheless, a noticeable gap still exists and grows with increasing ϕ̅_2_. As a result, annealing produces overall greater magnification ratios in cylinder radius than quenching, but still falls short of achieving the ideal equilibrium ratios predicted by SCFT.

To quantitatively assess how closely each structure-formation process approaches equilibrium, we evaluate the number density of cylindrical domains by dividing the number of domains within the simulation box by the box area. This serves as an effective metric, capturing the combined effects of lattice spacing and defectivity in the cylinder arrangement. In this analysis, we do not exclude highly distorted domains (see section Post-Processing of Density Data from Simulations of the SI), as such distortions arise from connections between adjacent cylinders and thus represent a form of defectivity reflected by a reduced domain count. Figure 9 presents the time evolution of this quantity for several representative simulations selected at γ_2_ = 2.25, along with the equilibrium number density of domains predicted by SCFT. In all cases, the number density of domains decreases over time due to cylinder-fusion events, but this decrease rapidly slows down and the cylinder density remains significantly higher than the equilibrium prediction for the entire simulation duration. The percentage differences between the simulated and equilibrium domain densities at *t* = 12τ_*R*_ are compared in Figure 10. With identical processing condition, i.e., quenching, the percentage difference in the cylinder number density between simulation and SCFT prediction increases with ϕ̅_2_. On the other hand, changing the processing condition from quenching to annealing brings the morphology only slightly closer to equilibrium. These observations can be rationalized by the larger length-scale difference at higher ϕ̅_2_, as discussed in Figure 8.

**Figure 9:**
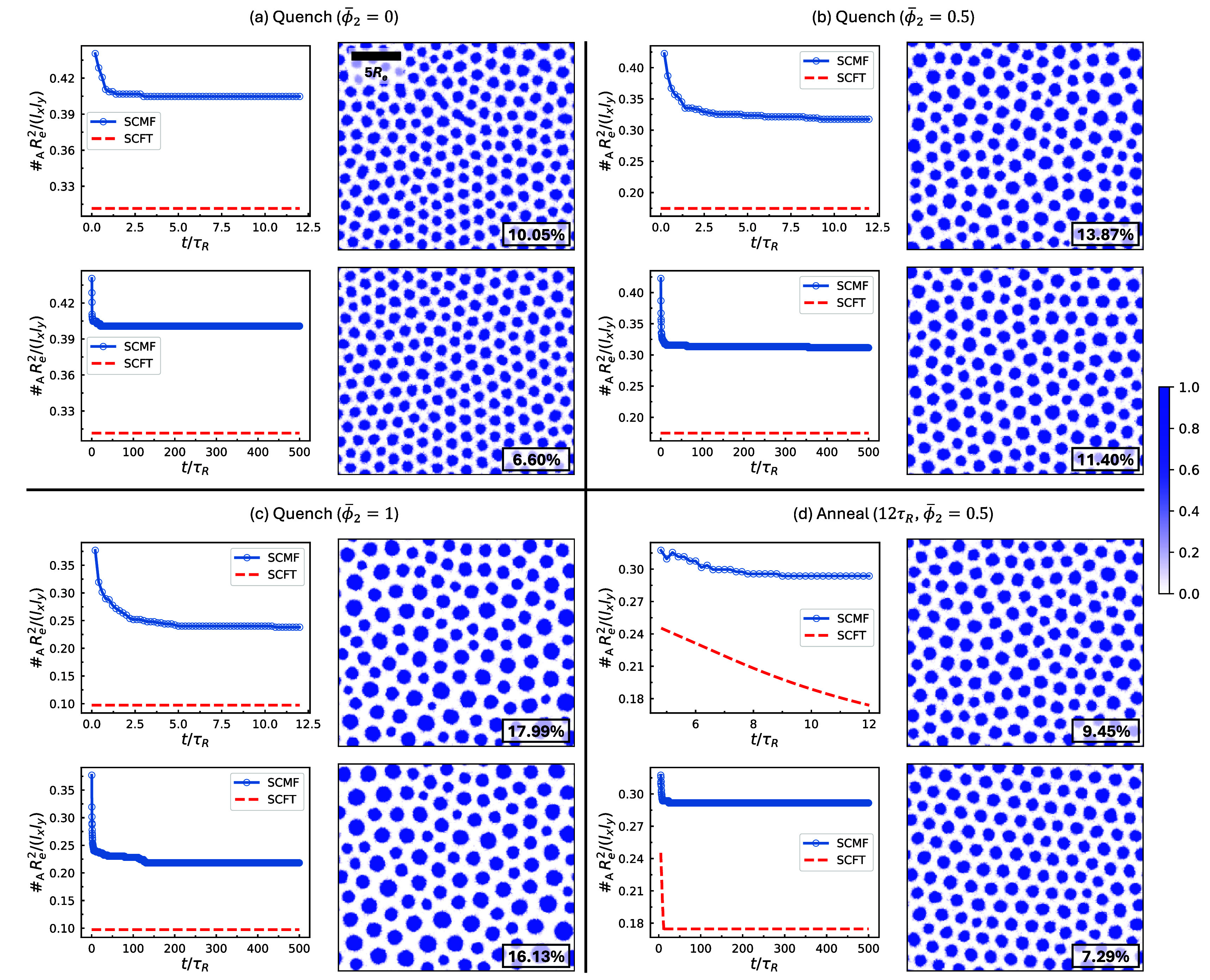
Time evolution of the number density of A-core cylinders for several representative simulations. The processing condition and ϕ̅_2_ are indicated at the top of each panel. Other parameters are *f*_1_ = *f*_2_ = 5/16, γ_2_ = 2.25, and χ_AB_ *N*_1_ = 30 (the target value for both quenching and annealing). In (d), annealing lasts for 12τ_*R*_. In each panel, the top row shows data up to 12τ_*R*_, while the bottom row extends the simulation to 500τ_*R*_. Dashed curves represent equilibrium values predicted by SCFT. To the right of each number-density plot, the corresponding normalized A-block density profile at the final time point is shown. For each density snapshot, the relative standard deviation (RSD) of cylinder radii is indicated in the bottom-right corner. Axis labels and ticks follow those in Figure 6 and are omitted here. A scale bar is included in the top density snapshot of panel (a) to indicate the length scale 5*R*_*e*_.

**Figure 10:**
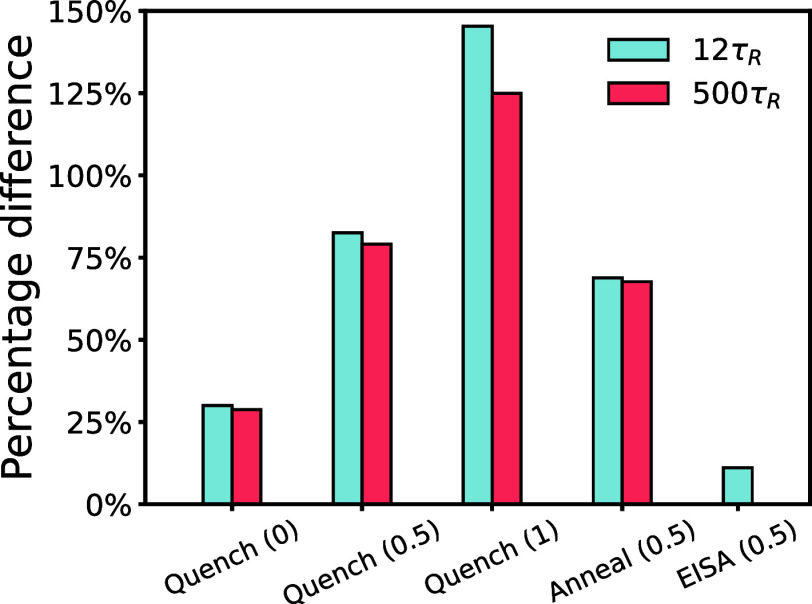
Relative difference between cylinder density obtained by SCMF simulations and the corresponding equilibrium values predicted by SCFT for different systems: binary copolymer melts (from Figure 9) and the EISA polymer solution analyzed in Figure 14d. The value in parentheses refers to ϕ̅_2_^bi^ for EISA and ϕ̅_2_ for all other cases.

In Figure 9, snapshots of the normalized A-block density at *t* = 12τ_*R*_ of the simulations are also presented, along with the corresponding relative standard deviation (RSD) of the cylinder radii. The cylinder radii resulting from quenching become increasingly polydisperse with rising ϕ̅_2_. In contrast, slow annealing yields cylinder radii with a significantly lower RSD. During annealing, χ_AB_ *N*_1_ gradually increases from the order–disorder-transition­(ODT) value, allowing the system sufficient time to form a cylindrical pattern with smaller and more uniform radii. However, as χ_AB_ *N*_1_ increases further, the system morphology becomes trapped into an initially established, metastable cylindrical structure, and subsequent cylinder fusion is a rare event with a high free-energy barrier. Thus, the metastable structure established in the early stage of structure formation persists, producing cylinders that are relatively monodisperse but whose average radius remains well below the equilibrium value.

To ensure that the results are not significantly affected by simulation time, selected simulations are extended from 12τ_*R*_ to 500τ_*R*_ (bottom row of each panel in Figure 9). Beyond 12τ_*R*_, additional fusion events occur occasionally; however, a substantial gap between the observed cylinder density and the equilibrium value persists. Compared to the snapshots at 12τ_*R*_, those at 500τ_*R*_ exhibit a narrower distribution of cylinder radii and improved HEX ordering. One exception is at ϕ̅_2_ = 1, where no clear HEX order is observed even at *t* = 500τ_*R*_. The marginal reduction in the relative difference between simulated and equilibrium cylinder densities from 12τ_*R*_ to 500τ_*R*_ (Figure 10) indicates that the system reaches a long-lived metastable state by 12τ_*R*_ and becomes effectively trapped. Overall, simulations at 500τ_*R*_ support the same conclusions as those at 12τ_*R*_. These results suggest that the magnification ratios predicted by SCFT are unlikely to be achieved through either quenching or annealing, as neither condition drives the system sufficiently close to equilibrium.

#### Evaporation-Induced Self-Assembly (EISA)

Another common method to induce structure formation in block copolymer films and porous membranes is evaporation-induced self-assembly (EISA). In EISA, polymers are initially dissolved in one or more solvents to form a disordered solution. As the solvent(s) evaporate, the polymer concentration near the solution surface increases (skin formation) and eventually surpasses the spinodal of the DIS structure, triggering structure formation. Under suitable structural, thermodynamic and processing conditions (e.g., polymer–solvent interactions, polymer–gas interactions, and evaporation rate), cylindrical domains oriented perpendicular to the solution surface form.^43,44,57^ We now investigate how effectively the size of these perpendicular cylinders formed via EISA can be adjusted by blending in a second, longer copolymer. In particular, we consider the same binary diblock copolymer blends used in the quenching and annealing simulations, with *f*_1_ = *f*_2_ = 5/16, γ_2_ = 1.5 or 2.25, and χ_AB_ *N*_1_ = 30. In addition to the two diblock copolymers, the EISA solution comprises a volatile solvent (S) and a nonvolatile solvent (C) – a formulation commonly used to fabricate integral asymmetric isoporous membranes.^8^ To simulate EISA, a large three-dimensional (3D) simulation cell with dimensions 24 × 21 × 50 *R*_*e*_^3^ is used because of the variation of the solvent densities deep inside the casting solution (*vide infra* Figure 14). A dynamic conversion zone is implemented, where the volatile solvent molecules are converted to gas (G) molecules (see section Particle-Based Monte Carlo Simulation of the SI for a description of the simulation setup).

The morphological evolution during EISA is highly sensitive to the interactions among the different components,^57^ characterized by a matrix of χ_αβ_ *N*_1_ parameters. We adopt the following interaction matrix:
1

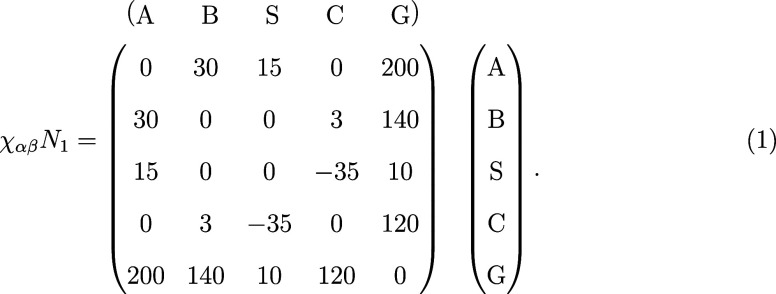

These interaction parameters have been established in a previous study to promote the formation of perpendicular cylinders under solvent evaporation of a casting solution containing monodisperse diblock copolymers with *f*_1_ = 5/16 and initial average concentrations ϕ̅_P_^(0)^ = ϕ̅_S_^(0)^ = ϕ̅_C_^(0)^ = 1/3.^44^

At the beginning of EISA, the casting solution must be (meta)­stable. In the ϕ̅_P_–ϕ̅_S_–ϕ̅_C_ Gibbs triangles in Figure 11, we present the spinodals of the various casting solutions evaluated using RPA. The ϕ̅_P_ axis of the Gibbs triangle denotes the total average concentration of the copolymers, i.e., ϕ̅_P_ = ϕ̅_1_ + ϕ̅_2_, and the volume fraction of the A_2_B_2_ copolymers relative to the total polymer content ϕ̅_2_^bi^ = ϕ̅_2_/(ϕ̅_1_ + ϕ̅_2_) is indicated in the legend. For ϕ̅_2_^bi^ = 0, the solution with ϕ̅_P_^(0)^ = ϕ̅_S_^(0)^ = ϕ̅_C_^(0)^ = 1/3 is clearly located within the (meta)­stable region. As A_2_B_2_ copolymers are blended into the solution, ϕ̅_2_^bi^ > 0, the overall segregation increases, shifting the spinodal toward lower ϕ̅_P_. To ensure that the homogeneous casting solution remains stable, we use the following simple procedure to adjust ϕ̅_P_^(0)^: First, we identify the intersections between the spinodal curves and the path ϕ̅_S_ = ϕ̅_C_ (denoted as ϕ̅_P_^*^) for all solutions. Second, we choose ϕ̅_P_^(0)^ for ϕ̅_2_^bi^ > 0 such that the difference between ϕ̅_P_^(0)^ and ϕ̅_P_^*^ matches that in the case ϕ̅_2_^bi^ = 0. The values of ϕ̅_P_^(0)^ determined for different solutions are shown as solid circles in Figure 11.

**Figure 11:**
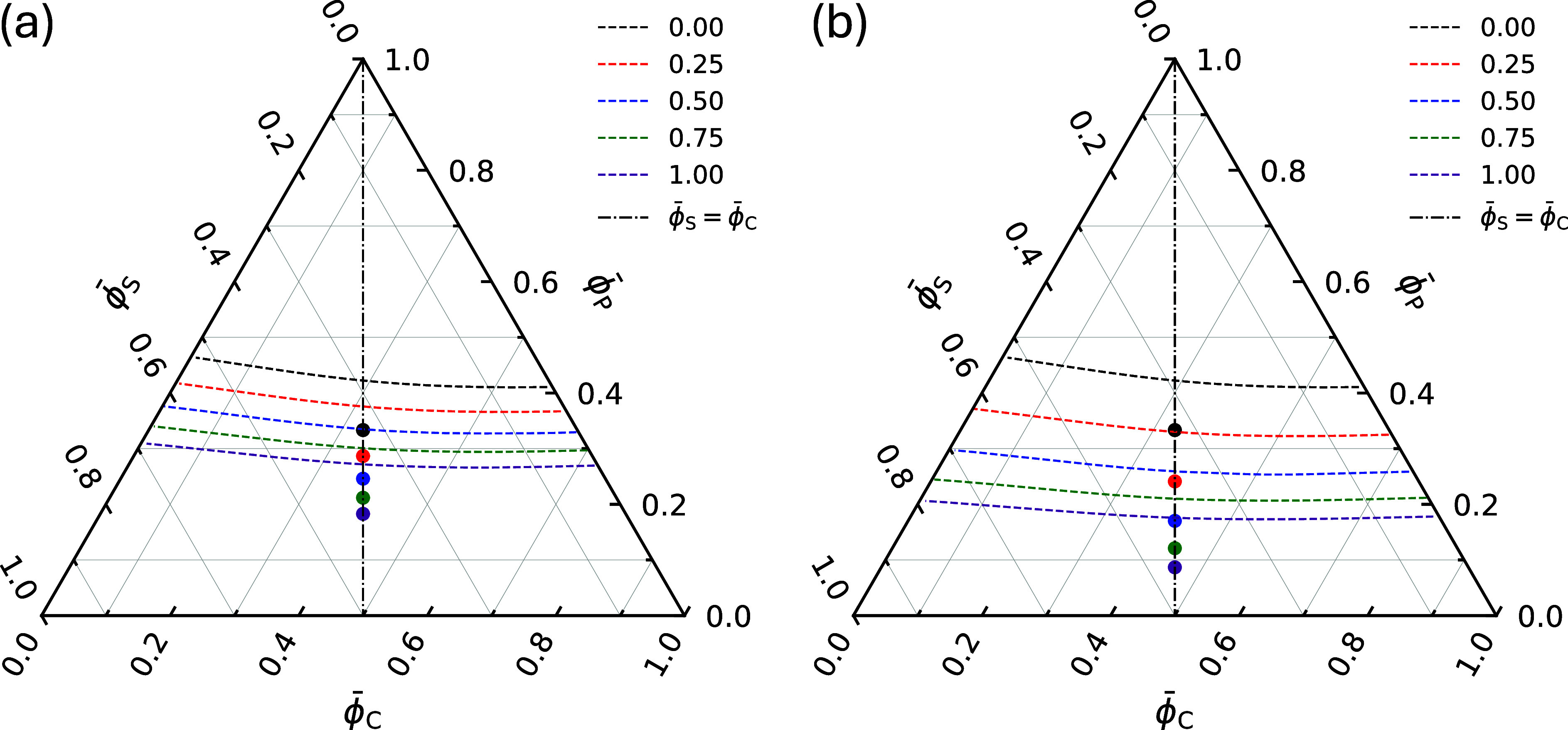
Gibbs triangles for casting solutions used in the SCMF simulations for EISA at *f*_1_ = *f*_2_ = 5/16 with γ_2_ = (a) 1.5 and (b) 2.25. The interaction parameters χ_αβ_ *N*_1_ are given in eq 1. Dashed lines indicate spinodal boundaries for different values of ϕ̅_2_^bi^. The disordered structure is unstable above the spinodal lines and (meta)­stable below. Solid circles mark the initial blend compositions of the casting solutions. The dash-dotted line marks the ϕ̅_S_ = ϕ̅_C_ isopleth.

To illustrate the structure formation during EISA, Figure 12 presents snapshots of the A-block density for γ_2_ = 1.5 and ϕ̅_2_^bi^ = 0.5 at several representative times. At *t* = 0τ_*R*_, the system is initialized in the disordered casting solution. As solvent evaporation progresses, the solution surface recedes downward, beneath which a thin layer of perpendicular A-rich cylinders begins to form. This layer gradually thickens over the course of EISA, which lasts for a total duration of 12τ_*R*_. Simulation snapshots of the A-block density at the end of the EISA process, 12τ_*R*_, for polymer solutions with different ϕ̅_2_^bi^ are shown in Figure 13. As ϕ̅_2_^bi^ increases, the number of domains formed during EISA clearly decreases, indicating an increase in the intercolumnar spacing in the top layer.

**Figure 12:**
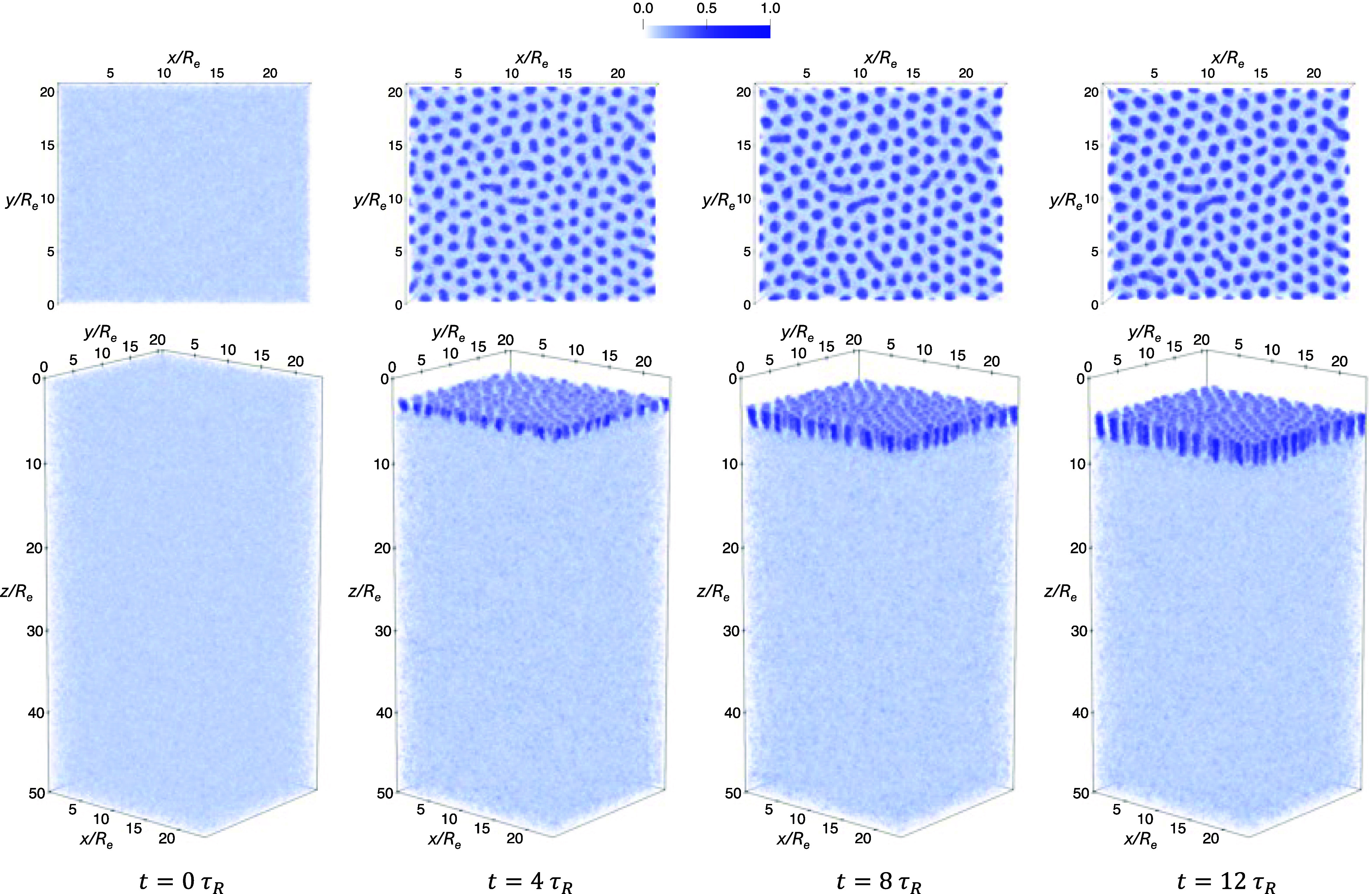
Normalized 3D density profiles at representative time points during an EISA simulation with γ_2_ = 1.5, ϕ̅_2_^bi^ = 0.5 and interaction parameters χ_αβ_ *N*_1_ given in eq 1. The top panel shows a top-down view of the corresponding morphology in the bottom panel.

**Figure 13:**
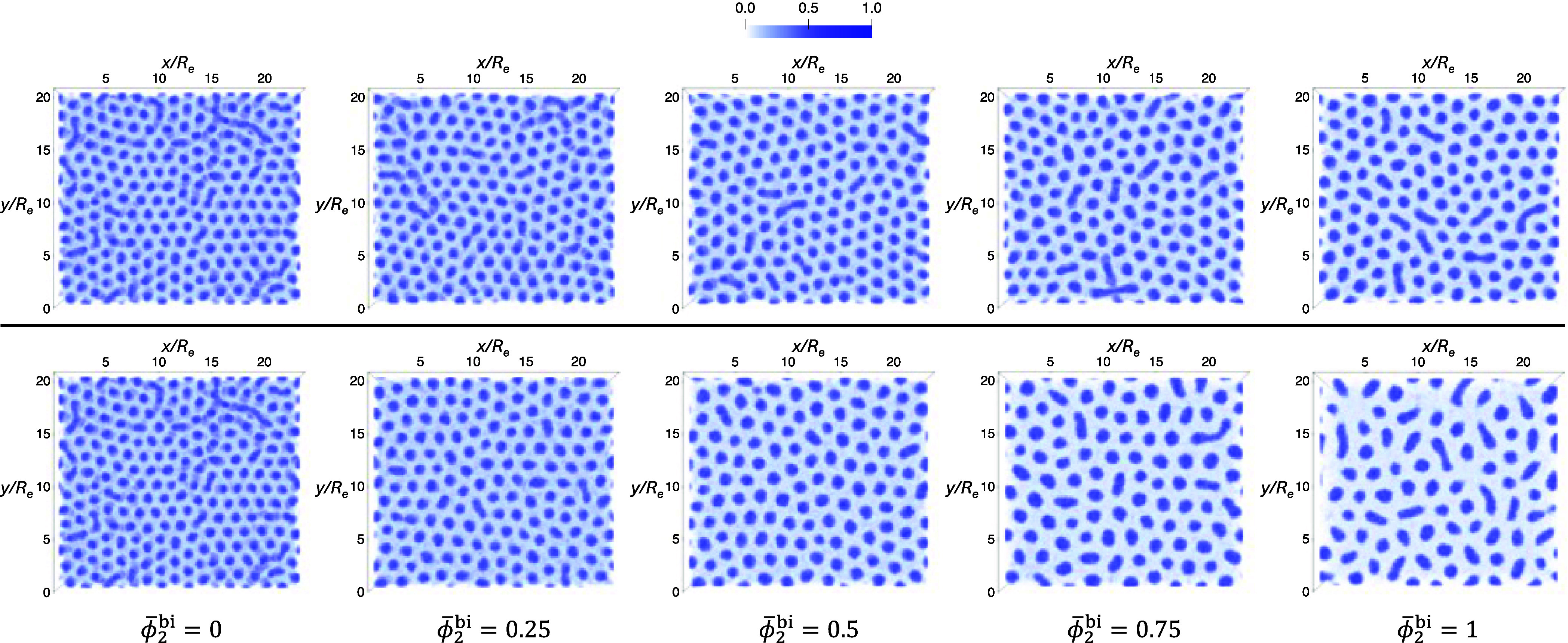
Top-down views of the morphologies at the end of EISA simulations (*t* = 12τ_*R*_), using casting solutions marked by solid circles in Figure 11. Top panel: γ_2_ = 1.5; bottom panel: γ_2_ = 2.25. The value of ϕ̅_2_^bi^ for each morphology is indicated below each column.

The data at ϕ̅_2_^bi^ = 1 and the larger asymmetry γ_2_ = 2.25 are omitted in the following analysis because of the small number of well-defined cylinders at *t* = 12τ_*R*_. The larger molecular weight increases the free-energy barriers for morphological changes, and the EISA duration, measured in units of the relaxation time of the long A_2_B_2_ copolymers, is rather short.

Although the presence of solvents alters the absolute values of the equilibrium domain spacing, *d*_c_, and cylinder radius, *r*_c_, in the HEX phase, precluding direct comparison with solvent-free melts, the magnification ratios can still be meaningfully compared. The magnification ratios of cylinder radii determined from EISA simulations for different casting solutions are presented as green open diamonds in Figure 7 (see section Extraction of Average Cylinder Radii from EISA Simulations of the SI for details on measuring the average cylinder radius from EISA simulations). Due to the high computational cost of the large-scale EISA simulations, replicate runs are performed only at ϕ̅_2_ = 0.5 for each γ_2_ to estimate the statistical uncertainty. Notably, the magnification ratios obtained from the EISA process consistently exceed those from both quenching and annealing processes of the corresponding binary copolymer melts. For γ_2_ = 1.5, the blending-induced magnification ratios in cylinder size achieved via EISA is comparable to the equilibrium values predicted for binary melts. A similar trend is observed for γ_2_ = 2.25, particularly at ϕ̅_2_^bi^ = 0.25 and 0.5, where the ratios achieved via EISA even surpasses the equilibrium values of the corresponding binary melts. Taken together, these results demonstrate that incorporating a second, longer copolymer in EISA leads to greater cylinder-size magnification than quenching or annealing the binary blends in their molten state.

Compared to quenching or annealing processes in binary diblock copolymer melts, the EISA process exhibits greater complexity. The laterally averaged densities of the components in an EISA simulation with *f*_1_ = *f*_2_ = 5/16, γ_2_ = 2.25, and ϕ̅_2_^bi^ = 0.5 at different times are presented in Figure 14a–c. As the S solvent evaporates, the solution surface shifts toward larger *z* values, resulting in the formation of a polymer skin near the liquid–gas interface. Within the top layer, the long A_2_B_2_ copolymers exhibit larger enrichment than the A_1_B_1_ chains, likely due to the slower diffusion of the longer chains and their concomitantly larger Péclet number. At the interface between this polymer skin and the remaining disordered casting solution, a depletion zone of A_2_B_2_ chains emerges. This suggests that the longer chains, originating from the casting solution, preferentially accumulate in the polymer skin. To rationalize this behavior, we compute the chemical potentials of the disordered casting solution in the semigrand canonical ensemble based on its composition. Using these chemical potentials, we then perform semigrand canonical SCFT calculations to determine the average concentrations of the HEX phase: ϕ̅_1_ = 0.0346, ϕ̅_2_ = 0.8051, ϕ̅_S_ = 0.0828, ϕ̅_C_ = 0.0775. The equilibrium concentration of A_2_B_2_ copolymers in this HEX phase is approximately an order of magnitude higher than in the homogeneous, metastable, casting solution. The observed migration of A_2_B_2_ chains from the casting solution into the polymer skin can be explained by the fact that ϕ̅_2_ in the nonequilibrium skin is smaller than in the HEX phase at the chemical potentials of the casting solution. Overall, Figure 14a–c reveal nontrivial density profiles for the various components during the EISA process.

**Figure 14:**
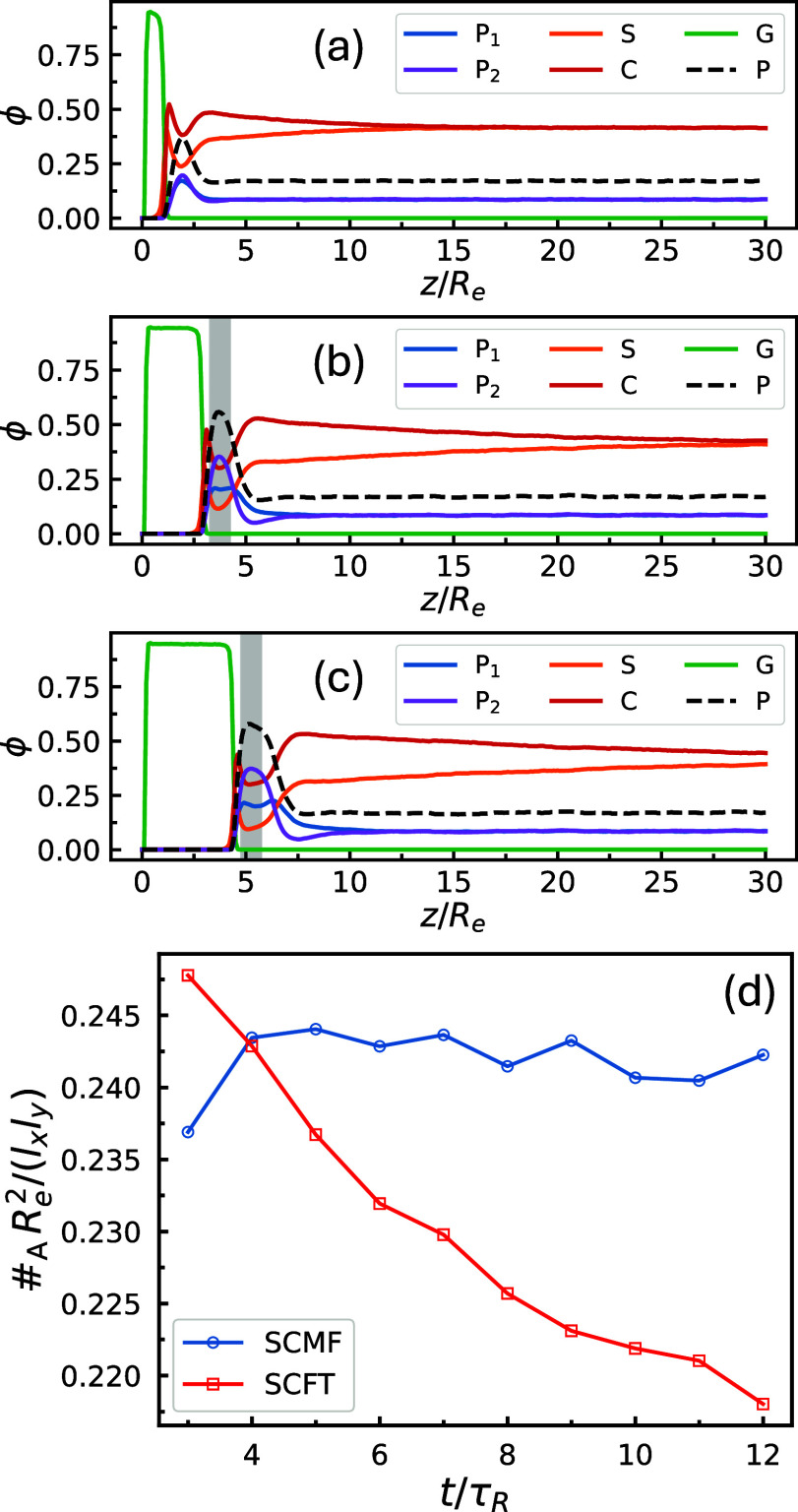
Laterally averaged one-dimensional (1D) density profiles of various components along the *z* direction from an EISA simulation with *f*_1_ = *f*_2_ = 5/16, γ_2_ = 2.25, and ϕ̅_2_^bi^ = 0.5, shown at *t* = (a) 1τ_*R*_, (b) 6τ_*R*_, and (c) 12τ_*R*_. Here, ϕ_P1_(**r**) = ϕ_A1_(**r**) + ϕ_B1_(**r**), ϕ_P2_(**r**) = ϕ_A2_(**r**) + ϕ_B2_(**r**), and ϕ_P_(**r**) = ϕ_P1_(**r**) + ϕ_P2_(**r**). To focus on the top layer, only data from 0 to 30*R*_*e*_ are shown, out of a total depth of 50*R*_*e*_. Panel (d) shows the time evolution of the average domain number density from the same EISA simulation. Data points are obtained by tracking a thin layer of thickness 1*R*_*e*_ centered around the *z* position of maximal, laterally averaged polymer concentration during EISA, and averaging over slices within this layer. In panels (b) and (c), the *z* ranges defining this thin layer are shaded in gray. Equilibrium values of the average domain number density predicted by SCFT, based on the instantaneous, laterally averaged concentrations within the same layer, are also shown for comparison.

Figure 14d presents the time evolution of the average cylinder density from the EISA simulation shown in Figure 14a–c. At each time point, the average cylinder density is calculated by averaging over slices within a thin layer of thickness 1*R*_*e*_ (e.g., gray-shaded *z* ranges in Figure 14b,c), centered at the *z* position corresponding to the maximum laterally averaged polymer concentration. For comparison, the corresponding instantaneous equilibrium values predicted by SCFT, based on the normalized average concentrations within this thin layer, are also presented. In contrast to all cases in Figure 9, by the time clearly identifiable cylindrical domains emerge (*t* = 3τ_*R*_ in Figure 14d), the measured cylinder density is already close to the equilibrium prediction. During the subsequent evaporation of solvent S, the equilibrium cylinder density decreases only slightly, resulting in minimal deviation between the measured value at the end of the EISA process and the corresponding equilibrium value. The relative difference between the simulated domain count at *t* = 12τ_*R*_ and the equilibrium prediction is compared with those for the quenching and annealing simulations in Figure 10, where this deviation is notably smaller for EISA than for all other processes.

The EISA process enables the system to rapidly approach the instantaneous equilibrium state during the early stage of structure formation, as evidenced in Figure 14d. The effect of solvent evaporation is 2-fold: (i) Similar to annealing, solvent dilution initially leads to a lower segregation strength and, consequently, a larger fastest-growing length scale during the early stage of spinodal decomposition compared to quenching. This narrows the gap between the characteristic length scale of the emerging morphology and that of the instantaneous equilibrium structure. By the same token, this leads to the formation of cylinders with more uniform radii during EISA. (ii) Solvent dilution additionally gives rise to a smaller initial value of the invariant degree of polymerization, $\overset{¯}{N}$ , thereby reducing the scale $\sqrt{N®}k_{B}T$ of free-energy barriers and facilitating structural ordering. Subsequently, segregation strength and $\overset{¯}{N}$ gradually increase during skin formation.

The first point is demonstrated in Figure 8c, where the fastest-growing and instantaneous equilibrium length scales during EISA are compared at various ϕ̅_2_^bi^. To evaluate the fastest-growing length scale, we first compute the time-dependent concentrations averaged over a thin layer of thickness 1*R*_*e*_, centered at the *z* position of maximal polymer density. We linearly interpolate these concentrations in time, identify the spinodal, and determine the fastest-growing spinodal mode by RPA. The equilibrium length scale is estimated by the SCFT-predicted *d*_c_, using the same average concentrations associated with the magnification ratios in Figure 7. One can appreciate that EISA leads to evidently smaller length-scale differences, which the system has to overcome by coarsening. This observation accounts for the small deviation of the simulated cylinder density from the equilibrium value shown in Figure 10. Interestingly, Figure 8c also reveals that the length-scale gap resulting from EISA exhibits a slower increase with ϕ̅_2_^bi^, potentially contributing to the enhanced scaling of the magnification ratios observed in Figure 7.

As suggested by Figures 10 and 8c, we speculate that the magnification ratios observed in the EISA simulations (Figure 7) arise from two aspects: (1) the EISA process provides a pathway for the system to approach its instantaneous equilibrium state more closely; and (2) the equilibrium magnification ratios in cylinder radius achieved at the end of EISA (*t* = 12τ_*R*_) as a function of ϕ̅_2_^bi^ – accounting for the presence of S and C solvents – remain comparable to those in binary block copolymer melts. To verify this, we perform SCFT calculations to determine the magnification ratios at thermodynamic equilibrium, using the normalized average concentrations within the same *z* ranges over which the average radii in Figure 7 are computed. The results are presented in Figure 15, alongside the SCFT-predicted ratios for diblock copolymer melts and the corresponding values measured directly from the EISA simulations shown in Figure 7. For both γ_2_ = 1.5 and 2.25, the SCFT predictions (gray dashed curves) and direct measurements from the EISA simulations show excellent agreement, and both deviate only marginally from the SCFT-predicted values for the solvent-free melts (black dashed curves). This provides direct evidence that the magnification-ratio scaling achieved by EISA closely matches that obtained from its instantaneous structures.

**Figure 15:**
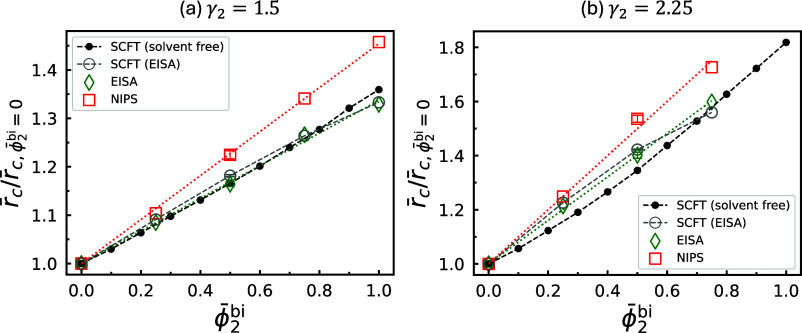
Magnification ratios of cylinder radii predicted by SCFT, based on normalized average concentrations extracted from EISA simulations (gray), and magnification ratios of pore radii measured from NIPS simulations (red), shown for (a) γ_2_ = 1.5 and (b) γ_2_ = 2.25. The *z* ranges used for extracting average concentrations from the EISA simulations match those used for evaluating the average radii in Figure 7. For comparison, the SCFT-predicted ratios for diblock copolymer melts and the corresponding values measured directly from the EISA simulations in Figure 7 are also reproduced. For solvent-free data points, ϕ̅_2_^bi^ reduces to ϕ̅_2_. Dotted lines represent linear fits.

### NIPS: Pore Sizes and Membrane Morphologies

Blending binary diblock copolymers provides an effective strategy to tune structure sizes for a variety of practical applications. A prominent example is the fabrication of integral asymmetric isoporous membranes via the two-step SNIPS process, which combines EISA with NIPS. During NIPS, the membrane is immersed in a nonsolvent bath, triggering solvent–nonsolvent exchange. The nonsolvent penetrates the self-assembled top layer through the A-rich cylinders, creating pores suitable for ultrafiltration. Subsequently, macrophase separation between the copolymers and the nonsolvent gives rise to a macroporous substructure beneath the top layer, providing mechanical support. As the process progresses, the structure becomes kinetically arrested when the dense top layer vitrifies. The final pore size of the membrane is closely related to the cylinder radius formed during EISA.

In this section, we perform SCMF simulations to quantitatively investigate how the addition of A_2_B_2_ copolymers influences both the membrane pore size and the morphological characteristics of the underlying macroporous substructure. Our goal is to identify the fundamental factors that define the upper limit of pore-size magnification in binary diblock copolymer blends processed via SNIPS, and to derive design principles for maximizing this tunability.

To initiate NIPS, all gas molecules present at the end of EISA (*t* = 12τ_*R*_) are converted into nonsolvent molecules. The nonsolvent interacts with the other segment types with strengths χ_NA_ *N*_1_ = 10, χ_NB_ *N*_1_ = 150, and χ_NS_ *N*_1_ = χ_NC_ *N*_1_ = −30.^44^ During NIPS, both S and C solvents are converted into nonsolvent within the conversion zone, which remains fixed at the position reached at the end of EISA. All NIPS simulations last for 18τ_*R*_ (from *t* = 12τ_*R*_ to 30τ_*R*_). Figure 16 presents an example of the normalized total polymer-density distribution at representative times during NIPS with γ_2_ = 1.5 and ϕ̅_2_^bi^ = 0.5. At *t* = 12τ_*R*_, the polymer concentration gradient established during EISA is clearly manifested as a color gradient along the *z* direction. As the nonsolvent, N, penetrates the dense top layer, it opens pores (with low total polymer density) inside the A-cores of the self-assembled cylinders. Subsequently, it rapidly undergoes macrophase separation from the polymer in the casting solution beneath, generating a spongy macroporous morphology. As the NIPS process progresses, the macrophase-separation front propagates downward, and the macroporous structure continues to grow. The structure above the front quickly vitrifies and remains largely unaltered at later times.

**Figure 16:**
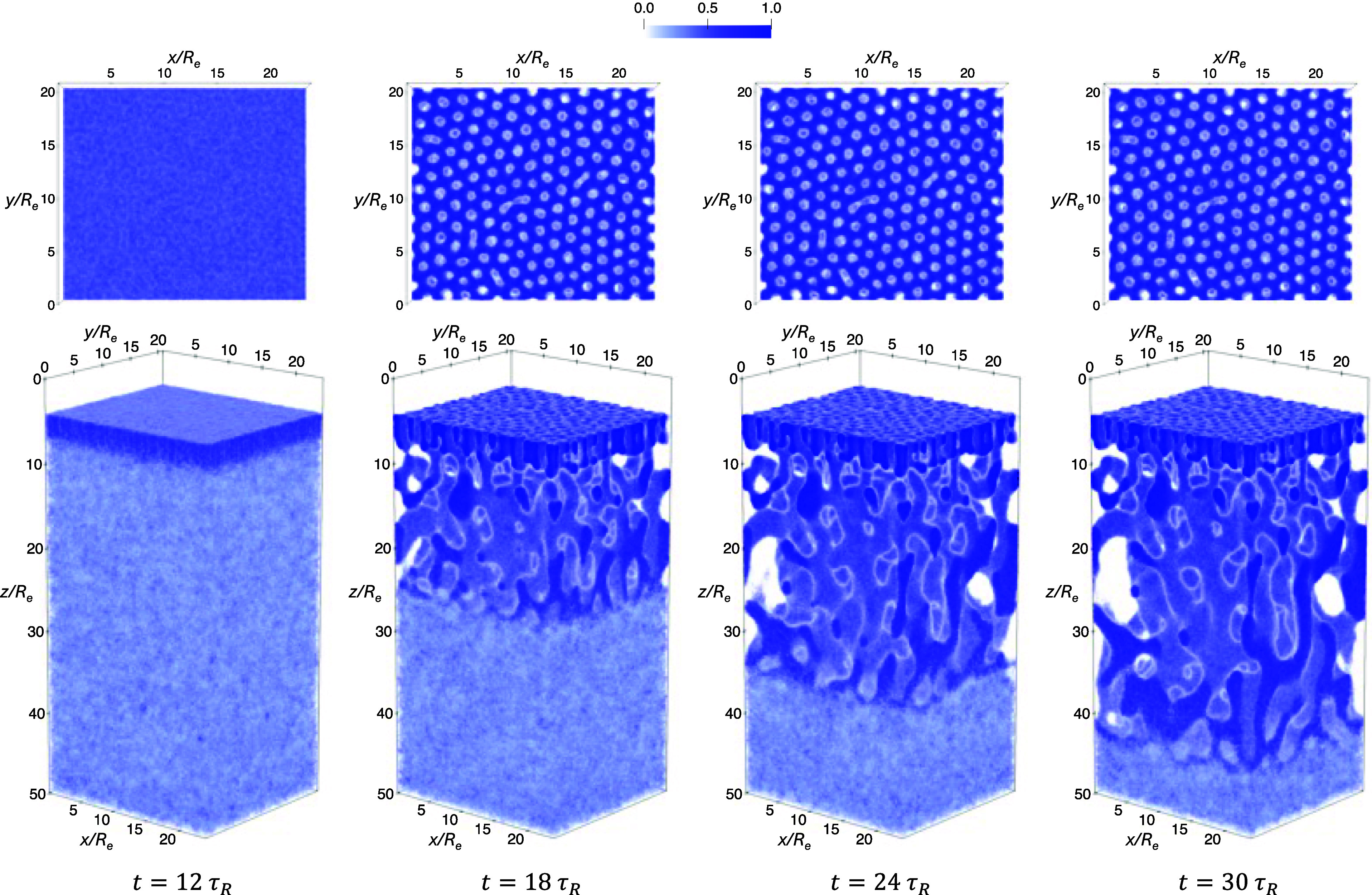
Normalized 3D density profiles at representative times during a NIPS simulation with γ_2_ = 1.5 and ϕ̅_2_^bi^ = 0.5. The top panel shows a top-down view of the corresponding morphology in the bottom panel.

Final simulation snapshots from the NIPS process at γ_2_ = 1.5 and γ_2_ = 2.25 are shown in Figures 17 and 18, respectively. In all cases, well-defined pores form within the top membrane layer, except for the case of γ_2_ = 2.25 at ϕ̅_2_^bi^ = 1, where an excessively high defect density is observed. Across all simulations, the liquid–gas interface retracts by a comparable distance over the same EISA duration (12τ_*R*_), reaching similar *z* positions, as evidenced by the similar top-surface locations in all panels. A lower polymer concentration in the casting solution (required for an initial, thermodynamically stable casting solution at higher ϕ̅_2_^bi^, see Figure 11) leads to reduced polymer accumulation during EISA, resulting in a thinner top layer. When this layer becomes too thin, it fails to maintain structural integrity during rapid nonsolvent intrusion in the NIPS process, thereby disrupting the formation of an ordered self-assembled morphology. This mechanism explains the high defect density observed at γ_2_ = 2.25 and ϕ̅_2_^bi^ = 1. For all other simulations, at higher ϕ̅_2_^bi^, the reduced total polymer concentration and the slower defect-annihilation dynamics, due to the increased fraction of longer A_2_B_2_ copolymers, cause a slightly higher defect density—especially at ϕ̅_2_^bi^ ≥ 0.75—yet the membrane pores remain largely well separated and relatively monodisperse in size.

**Figure 17:**
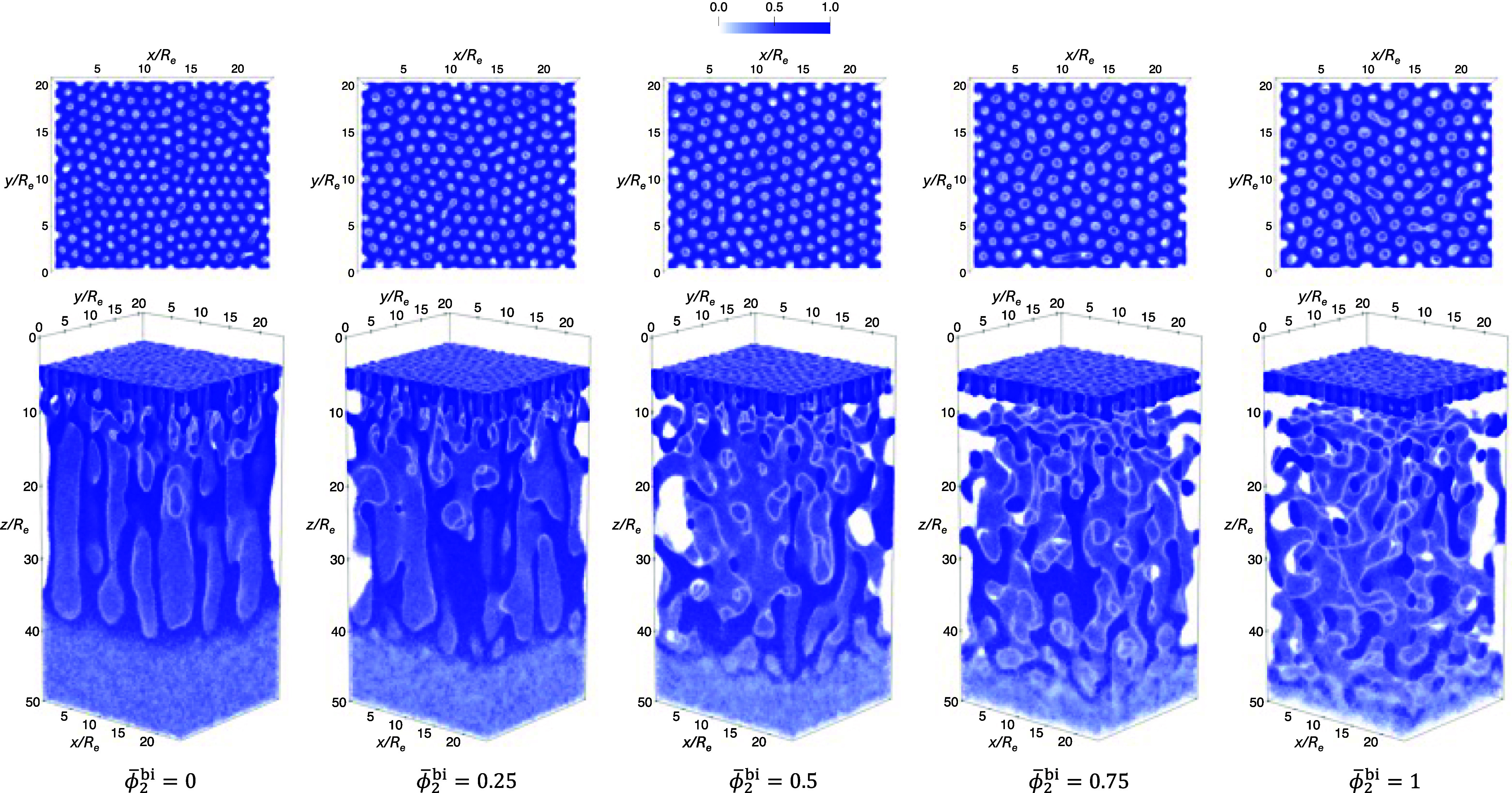
Normalized 3D density profiles at the end of NIPS simulations for γ_2_ = 1.5 and different ϕ̅_2_^bi^ values, following the EISA simulations in the top row of Figure 13. The top panel shows a top-down view of the corresponding morphology in the bottom panel.

**Figure 18:**
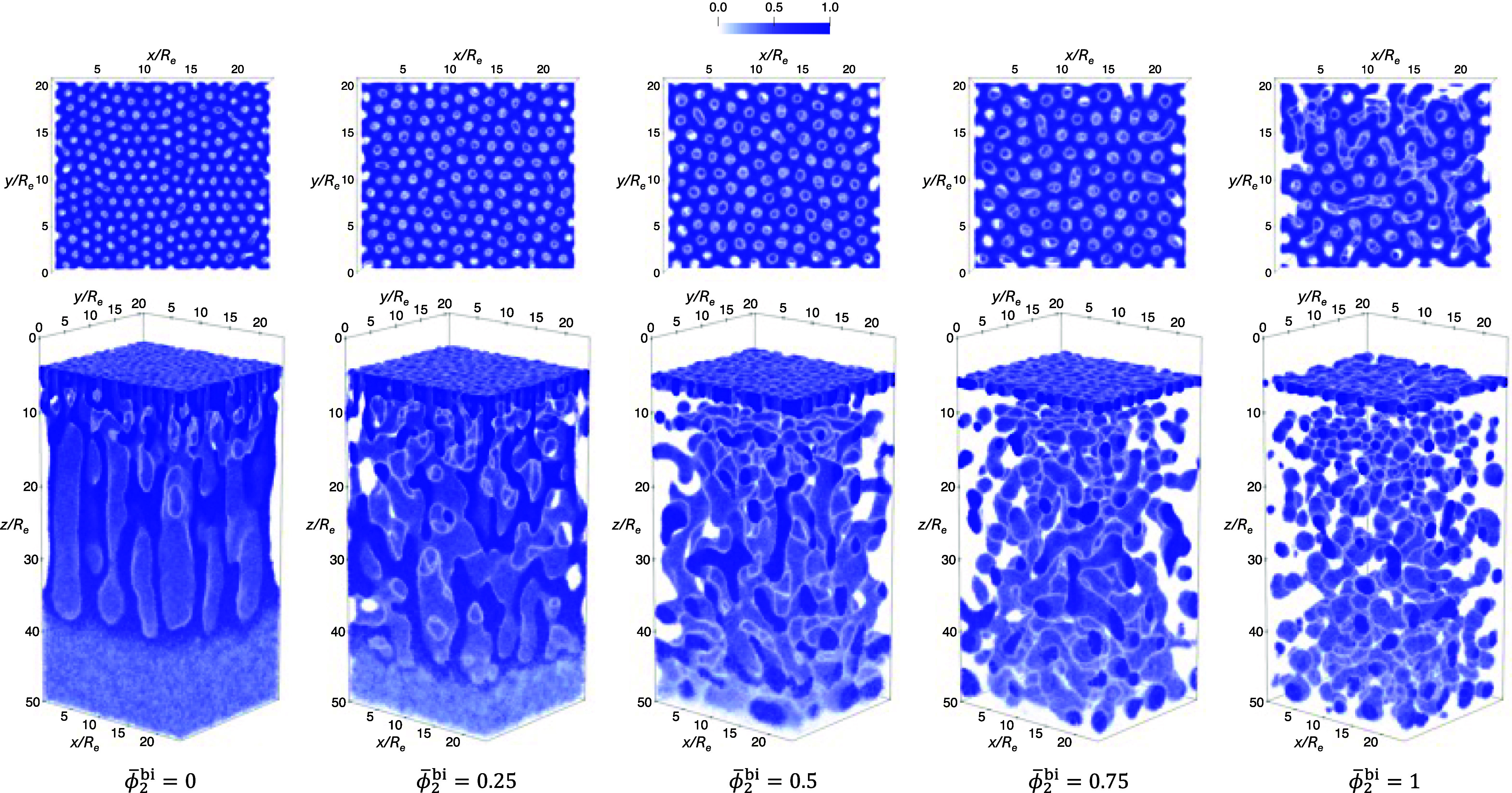
Normalized 3D density profiles at the end of NIPS simulations with γ_2_ = 2.25 and different ϕ̅_2_^bi^ values, following the EISA simulations in the bottom row of Figure 13. The top panel shows a top-down view of the corresponding morphology in the bottom panel.

To quantify the magnification ratio of the pore size, an effective pore radius is determined for each NIPS simulation at *t* = 30τ_*R*_ by analyzing the top layer of the membrane. For every *x*–*y* cross section at fixed *z*, pores are identified as regions where ϕ_N_(**r**) > ϕ_P_(**r**), and the average pore radius is computed following the postprocessing procedure described previously (see section Post-Processing of Density Data from Simulations of the SI). As one moves downward from the liquid–gas interface, the average pore radius initially decreases, reaching a minimum before gradually increasing. This minimum represents the narrowest constriction that governs filtration selectivity, and is defined as the effective pore radius of the membrane. The corresponding magnification ratios, calculated from these values, are shown in Figure 15. Similar to the values measured for cylinder radius from the EISA process, those measured for pore radius from NIPS exhibit a nearly linear dependence on ϕ̅_2_^bi^. Linear fitting reveals that the slope of the magnification ratio for the post-NIPS pore radius increases by approximately 35% and 25% relative to that of the pre-NIPS (aka post-EISA) cylinder radius for γ_2_ = 1.5 and 2.25, respectively.

The addition of A_2_B_2_ copolymers into the SNIPS process markedly influences the morphology of the membrane substructure. Notably, a polymer depletion zone between the self-assembled top layer and substructure emerges as ϕ̅_2_^bi^ increases. At high ϕ̅_2_^bi^, this gives rise to a complete gap in the polymer density, making the microporous top layer detached from the mechanically supportive substructure. Moreover, for γ_2_ = 2.25 and ϕ̅_2_^bi^ ≥ 0.75 in Figure 18, the polymer domains in the substructure exhibit weakened connectivity. The emergence of an overall disintegrated membrane structure severely undermines its mechanical stability, potentially leading to membrane collapse/densification.

Prior studies have shown that the morphology of SNIPS-fabricated membranes is influenced by a variety of structural, thermodynamic, kinetic, and processing parameters.^10,44,63,64^ While a comprehensive exploration of the structure/thermodynamics–processing–morphology relationship in SNIPS with two diblock copolymers would provide valuable guidance for membrane fabrication with finely controlled pore sizes, such a study lies beyond the scope of the present work and is deferred to future efforts. Nonetheless, we seek to optimize membrane morphology within a deliberately constrained parameter space, aiming to identify key conditions for preserving an integrated structure while finely tuning the pore size through the addition of A_2_B_2_ copolymers. To this end, we focus on the case with γ_2_ = 2.25, where the membrane integrity exhibits the most pronounced deterioration at high ϕ̅_2_^bi^.

We attribute the formation of a disintegrated membrane to two primary factors: First, at higher ϕ̅_2_^bi^, a lower ϕ̅_P_^(0)^ is required to stabilize the casting solution due to the increased degree of segregation, as shown in Figure 11. While necessary for maintaining solution stability, this results in insufficient polymer content to sustain connectivity between the top layer and the substructure. Moreover, the diminished polymer volume fraction compromises the structural integrity of the walls separating the finger-like perpendicular channels in the substructure, giving rise to an increasingly spongy morphologies observed in Figure 18 with decreasing ϕ̅_P_^(0)^. Second, in our simulations, the solvent and nonsolvent molecules are modeled as 8-bead oligomers. This parameter choice overestimates the relative mobility of the polymer chains compared to the small molecules, thereby exaggerating the formation of a disintegrated membrane structure prior to vitrification.

Motivated by the above considerations, we adopt a modified interaction matrix:

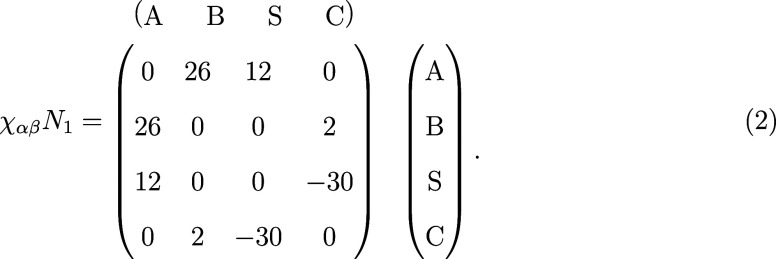

2
Compared to eq 1 (excluding the G species), the magnitudes of all nonzero entries are slightly reduced. This corresponds to a reduction in the overall segregation strength and can be experimentally realized by modifying the molecular chemistry, using smaller molecular weight, or elevating the temperature. The lower segregation degree broadens the (meta)­stability region of the disordered phase on the Gibbs triangle and is also expected to suppress the formation of disordered micelles,^44^ enabling a stable casting solution at higher ϕ̅_P_^(0)^. Furthermore, we increase the mobility asymmetry between the long polymer chains and small molecules by adjusting the MC update frequency. Specifically, the update frequency is reduced by a factor of 2 for A_1_B_1_ chains and by a factor of 4 for A_2_B_2_ chains to account for the lower mobility of longer polymer chains. This reduction mimics dynamic effects beyond the Rouse model, such as chain entanglement.

Using the interaction matrix in eq 2, the spinodal points along the ϕ̅_S_ = ϕ̅_C_ isopleth on the Gibbs triangle at different ϕ̅_2_^bi^ are shown as open black circles in Figure 19a. As expected, the spinodal curve shifts toward higher ϕ̅_P_ compared to the higher segregated system in Figure 11a. To determine the optimal values of ϕ̅_P_^(0)^ at various ϕ̅_2_^bi^, we employ a two-step procedure: First, we perform trial EISA simulations for several ϕ̅_P_^(0)^ to identify the values that yield perpendicular cylinders with minimal defects for the two limiting cases, ϕ̅_2_^bi^ = 0 and 1. Second, to avoid running simulations at every individual ϕ̅_2_^bi^, the ϕ̅_P_^(0)^ values for intermediate ϕ̅_2_^bi^ are estimated by interpolation. Two interpolation strategies are employed: (1) linear interpolation of ϕ̅_P_^(0)^ based on its values at the two limiting cases (method 1), (2) linear interpolation of the difference, Δϕ̅_P_^(0)^, between ϕ̅_P_^(0)^ and the spinodal point, ϕ̅_P_^*^, and ϕ̅_P_^(0)^ = ϕ̅_P_^*^ + Δϕ̅_P_^(0)^ (method 2). ϕ̅_P_^(0)^ values determined using these two methods are shown in Figure 19a.

**Figure 19:**
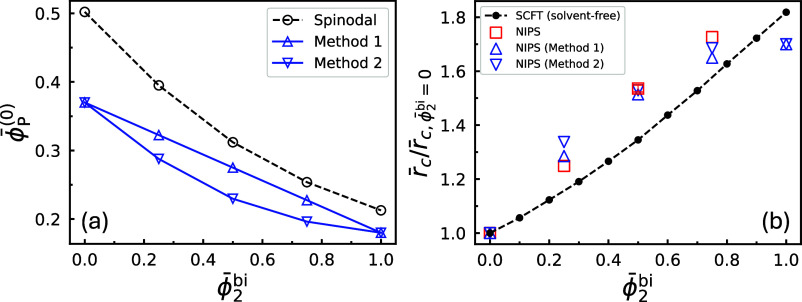
(a) Spinodal points and initial total polymer concentrations determined by two different methods at various ϕ̅_2_^bi^ values, for simulations with γ_2_ = 2.25, using optimized χ_αβ_ *N*_1_ parameters and MC update frequencies. For spinodal points, the ordinate axis corresponds to ϕ̅_P_. (b) Magnification ratios of pore radii measured in NIPS simulations with γ_2_ = 2.25, using optimized parameters and the two different estimates of ϕ̅_P_^(0)^ in (a). For comparison, the SCFT-predicted ratios for cylinder radii in diblock copolymer melts and the values measured for pore radii from NIPS simulations in Figure 15b are also reproduced in (b). For solvent-free data points, ϕ̅_2_^bi^ reduces to ϕ̅_2_.

The final morphologies from the modified simulations are depicted in Figures 20 and S20. With the parameter optimization, significant improvement of the morphological integrity of the membrane is observed. In contrast to the right-most panel in Figure 18, well-defined pores with much fewer defects can be achieved at ϕ̅_2_^bi^ = 1. Moreover, the membrane structures retain good connectivity upon increasing ϕ̅_2_^bi^, despite the morphological transition from finger-like to spongy. Figure S4 show enlarged snapshots from Figures 18, 20 and S3, highlighting the connection zones between the top layer and substructure. At ϕ̅_2_^bi^ = 1, a depletion zone remains visible in the optimized simulation, but moderate vertical connectivity is established between the top and bottom layers, markedly different from the complete gap observed in the top-right panel. For intermediate ϕ̅_2_^bi^ values, both interpolation methods yield good connectivity; however, method 2 produces cylindrical microporous channels in the top layer with better perpendicular alignment.

**Figure 20:**
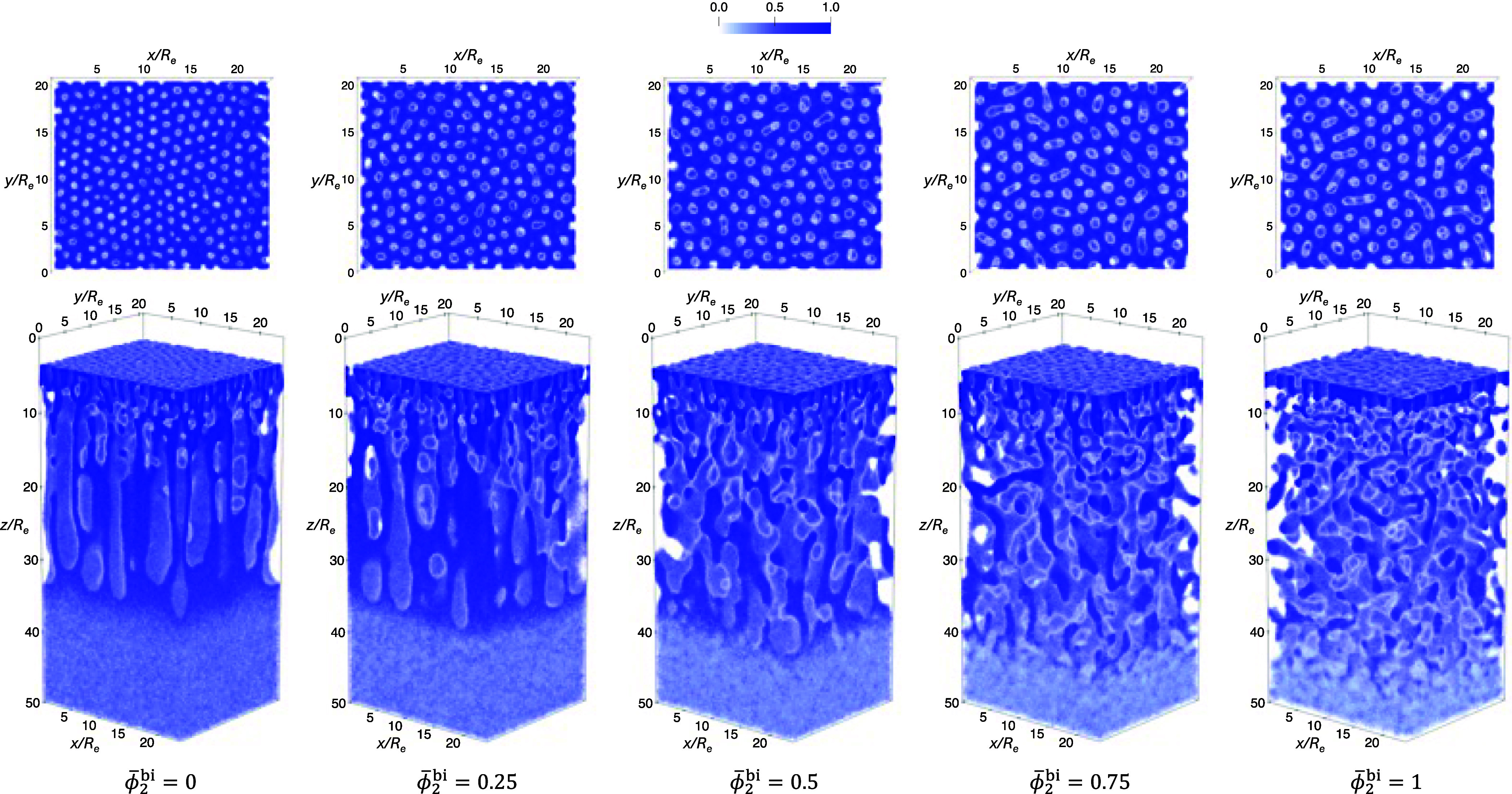
Normalized 3D density profiles at the end of NIPS simulations with γ_2_ = 2.25 and optimized parameters at different ϕ̅_2_^bi^ values. The ϕ̅_P_^(0)^ values are determined using method 1 described in the main text. The top panel shows a top-down view of the corresponding morphology in the bottom panel.

The pore-radius magnification ratios from the simulations with optimized parameters are compared to those from earlier simulations in Figure 19b. The ratios obtained from the optimized SNIPS simulations remain largely consistent with those from earlier simulations, indicating that the pore-size dependence on ϕ̅_2_^bi^ is robust. Despite this, a slight decrease in the slope of the magnification ratio with ϕ̅_2_^bi^ is observed. Notably, Figure 19b also suggests that, at ϕ̅_2_^bi^ = 1, the low mobility of the long A_2_B_2_ chains starts to cause deviations from the near-linear behavior in the magnification ratio defined by the first four data points. Additionally, as shown in Figures 17–20, and S3, the top layers overall exhibit increased defect densities at higher γ_2_ and ϕ̅_2_^bi^, compromising membrane selectivity. Further increases in γ_2_ would continue to impair both the magnification ratio and selectivity.

## Conclusions

In this work, we conducted a comprehensive investigation of the equilibrium and process-directed structure sizes in binary diblock copolymer blends, combining SCFT and particle-based simulations. Using SCFT, we constructed equilibrium phase diagrams and determined the optimal cylinder radii and intercolumnar distances. Within the explored parameter space, the cylinder radius of the equilibrium HEX phase can be continuously enlarged by up to 3-fold.

We further quantified the magnification of cylinder radii achieved by blending the long A_2_B_2_ copolymers under different processing conditions, including quenching, annealing, and EISA involving two – volatile and nonvolatile – solvents. As more A_2_B_2_ copolymers are added, the growing disparity in length scales that the blends must traverse in the course of coarsening prevents both quenching and annealing pathways from reaching equilibrium, resulting in smaller magnification ratios than predicted at equilibrium. In contrast, as ϕ̅_2_^bi^ increases, the EISA process exhibits a more ideal increase in the magnification ratio, closely approaching the equilibrium values predicted for solvent-free binary diblock copolymer blends.

Finally, we investigated the impact of incorporating A_2_B_2_ copolymers on the fabrication of integral-asymmetric isoporous membranes via the SNIPS process. Within the simulated parameter space, the incorporation of a second, longer A_2_B_2_ copolymer with γ_2_ = 2.25 allows a continuous pore size increase of up to 70% in the SNIPS-fabricated membranes. With further parameter tuning, we anticipate a more than 2-fold magnification to be feasible. For example, the composition *f*_1_ = 5/16 in our simulations lies near the upper boundary of HEX-stability. A possible strategy to expand the accessible magnification range is to select *f*_1_ near the lower boundary and *f*_2_(>*f*_1_) near the upper stability limit of the HEX phase, enabling broader pore-size tuning as ϕ̅_2_^bi^ varies between 0 and 1 (compared to the choice *f*_2_ = *f*_1_) while largely avoiding order–order transitions. However, the viable range of γ_2_ is ultimately constrained by declining membrane quality, arising from the lower ϕ̅_P_^(0)^ required to offset the enhanced segregation strength and the small mobility of the longer A_2_B_2_ chains at high γ_2_. Our simulations indicate that this limiting chain-length ratio, γ_2_, lies well below the threshold for macrophase separation between the two diblock copolymers.

Owing to the simple macromolecular architecture of the constituent copolymers, binary diblock copolymer blends offer a cost-effective yet versatile platform for stabilizing novel phases^58–62,65^ and fine-tuning structure lengths.^35,66,67^ Through an in-depth investigation of both equilibrium phase behavior and process-directed nonequilibrium morphologies, our work offers a deeper understanding of structure–processing–property relationships in these copolymer blends. These insights contribute to the foundation for the rational design of copolymer-based materials with finely tailored morphologies and structure dimensions, enabling future advances in applications such as high-performance filtration membranes and beyond.

## Supplementary Material



## Footnotes

1 The alternative mechanism of shrinking cylinders by transferring macromolecules from small to larger cylinders faces a significant single-chain free-energy barrier and is intrinsically slow.

2 The larger fastest-growing length scale during annealing compared to quenching, as shown in Figure 8, also helps explain the morphological characteristics observed in blends with higher γ_2_ discussed in Paper I.^39^ Specifically, for γ_2_ = 5 or 6, annealing produces cylindrical morphologies that exhibit a visibly larger average cylinder radius than those obtained by quenching. (see Figures 13 and S9 of Paper I^39^).

## Notes

Self-consistent field theory (SCFT); random-phase approximation (RPA); particle-based Monte Carlo simulation; additional figures; processing of simulation data: post-processing of density data from simulations; and extraction of average cylinder radii from EISA simulations (PDF)
