# Supplementary material for: Process-Directed Self-Assembly of Copolymer Blends: II. Continuous Tuning of Structure Size
Source: Macromolecules. 2025 Oct 24;58(21):11539–57. doi: 10.1021/acs.macromol.5c02630 (PMC12613822; doi:10.1021/acs.macromol.5c02630)
Supplement: Supplementary file 1 [file ma5c02630_si_001.pdf]

Supporting Information
for
Process-directed self-assembly of copolymer blends: II. Continuous tuning of structure size

Jiayu Xie* and Marcus Müller*

Institute for Theoretical Physics, Georg-August University of Göttingen, 37077 Göttingen, Germany

E-mail: jiayu.xie@theorie.physik.uni-goettingen.de; mmueller@theorie.physik.uni-goettingen.de

This PDF includes:

- Self-Consistent Field Theory (SCFT)
- Random-Phase Approximation (RPA)
- Particle-Based Monte-Carlo Simulation
- Additional Figures
- Processing of Simulation Data:
  - Post-Processing of Density Data from Simulations
  - Extraction of Average Cylinder Radii from EISA Simulations

Self-Consistent Field Theory (SCFT)

We describe the formulations of self-consistent field-theory (SCFT) and random-phase approximation (RPA) using the most complex system they address—the quaternary casting solution employed to fabricate porous membranes via evaporation-induced self-assembly (EISA) and nonsolvent-induced phase separation (NIPS). We assume the casting solution to be an incompressible system comprising n_1 short diblock copolymers (A_1B_1), n_2 long diblock copolymers (A_2B_2), n_S volatile solvent (S) molecules , and n_C nonvolatile solvent (C) molecules in a volume V. All molecular species are modeled as discrete Gaussian chains,[1] represented by coarse-grained beads connected by harmonic bonds. Each A_1B_1 copolymer consists of N_{A1} A beads and N_{B1} B beads, summing to a total of N_1 beads per chain. The same notation applies to A_2B_2 copolymers but with all subscripts "1" replaced by "2". The block fractions are therefore defined as $f_1 = f_{A1} = N_{A1}/N_1$ and $f_2 = f_{A2} = N_{A2}/N_2$. Each solvent molecule, S or C, is modeled as a short chain consisting of N_S or N_C beads, respectively. Throughout this work, we use the length N_1 of the short diblock copolymers (A_1B_1) as the reference and denote the ratio between the length of a chain of any molecular species i and this reference chain length by $\gamma_i = N_i/N_1$. In the quaternary system, i = 1, 2, S or C. In all theoretical calculations as well as particle-based simulations we set $N_1 = 64$ and $N_S = N_C = 8$. Different values of γ_2 (equivalently, N_2) are considered to investigate the effect of the copolymer chain-length ratio.

In SCFT and RPA calculations, we assume a uniform segment density ρ_0. The average concentration of molecular species i is given by $\bar{\phi}_i = n_i\gamma_i N_1/\rho_0 V$, where $\rho_0 V = \sum_i n_i\gamma_i N_1$ denotes the total number of beads in the system. We also assume that all bonds in the system have the same statistical segment length, b. The bonded potential for a single bond connecting segments t and $t+1$ is given by

$$\mathcal{H}_{\text{bond}} = \frac{3k_B T}{2b^2}|\mathbf{r}_{t+1} - \mathbf{r}_t|^2 \,. \tag{S1}$$

For a linear reference chain with N_1 beads and $N_1 - 1$ bonds, the statistical segment length, b, is related to the root-mean-squared end-to-end distance R_e of a noninteracting chain via $R_e = b\sqrt{N_1 - 1}$, which we choose as the unit of length.

We formulate the thermodynamics of the blend in the canonical ensemble, controlling the average concentrations, $\bar{\phi}_i$, of different molecular species. Within SCFT, the Helmholtz free-energy density can be written as:[2,3]

$$\begin{aligned}\frac{N_1 \mathcal{F}}{\rho_0 V k_{\mathrm{B}} T} = &- \sum_i \frac{\bar{\phi}_i}{\gamma_i} \ln \frac{Q_i}{\bar{\phi}_i} - \frac{1}{V} \int \mathrm{d}\mathbf{r} \; \Bigg[\sum_\alpha N_1 \omega_\alpha(\mathbf{r}) \phi_\alpha(\mathbf{r}) \\ &- \frac{1}{2} \sum_{\alpha,\beta(\neq\alpha)} \chi_{\alpha\beta} N_1 \phi_\alpha(\mathbf{r}) \phi_\beta(\mathbf{r}) + \eta(\mathbf{r}) N_1 \left(1 - \sum_\alpha \phi_\alpha(\mathbf{r}) \right) \Bigg] ,\end{aligned} \tag{S2}$$

where VQ_i is the single-chain partition function of molecular species i (including its translational entropy); $\phi_\alpha(\mathbf{r})$ denotes the density field of segment species α = A, B, S, or C; $\omega_\alpha(\mathbf{r})$ represents the auxiliary field conjugate to $\phi_\alpha(\mathbf{r})$; $\chi_{\alpha\beta}$ is the Flory-Huggins interaction parameter quantifying the incompatibility between components α and β; and $\eta(\mathbf{r})$ is the Lagrange field enforcing incompressibility.

Extremizing Equation S2 leads to the following SCFT equations:

$$\omega_\alpha(\mathbf{r}) = \sum_{\beta(\neq\alpha)} \chi_{\alpha\beta} \phi_\beta(\mathbf{r}) + \eta(\mathbf{r}), \tag{S3}$$

$$\phi_\alpha(\mathbf{r}) = \sum_i \frac{\bar{\phi}_i}{\mathcal{Q}_i N_i} \sum_{t=1}^{N_i} \delta_{\alpha_i(t),\alpha} \; q_i(t,\mathbf{r}) e^{\omega_{\alpha_i(t)}(\mathbf{r})} q_i^\dagger(t,\mathbf{r}) \tag{S4}$$

$$\sum_\alpha \phi_\alpha(\mathbf{r}) = 1. \tag{S5}$$

where $\alpha_i(t)$ specifies the segment type of the tth segment on molecule species i.

In Equation S4, $q_i(t,\mathbf{r})$ is the chain propagator for molecular species i, which is solved

by iterating the following equation:

$$q_i(t+1,\mathbf{r}_{t+1}) = e^{-\omega_{\alpha_i(t+1)}(\mathbf{r}_{t+1})} \int d\mathbf{r}_t \; p_{t+1,t}(\mathbf{r}_{t+1}-\mathbf{r}_t) q_i(t,\mathbf{r}_t), \tag{S6}$$

with the initial condition $q_i(1,\mathbf{r}) = e^{-\omega_{\alpha_i(t)}(\mathbf{r})}$ where $\alpha_i(t)$ is the type of segment $t = 1$ on molecular species i. The bond transition probability from the tth to $(t+1)^{\text{th}}$ beads takes the form:

$$p_{t+1,t}(\mathbf{r}_{t+1}-\mathbf{r}_t) = \left(\frac{3}{2\pi b^2}\right)^{\frac{3}{2}} \exp\left(-\frac{3|\mathbf{r}_{t+1}-\mathbf{r}_t|^2}{2b^2}\right). \tag{S7}$$

The function $q_i^\dagger(t,\mathbf{r})$ in Equation S4 is the adjoint propagator to $q_i(t,\mathbf{r})$. It is obtained by solving the same equation (Equation S6), but iterated in the opposite direction along the chain. Since the (homopolymer) solvents, $i =$ S and C are invariant under $t \to N_i - 1 + 1$, the adjoint propagators for S and C are given by $q_i^\dagger(t,\mathbf{r}) = q_i(N_i - t + 1, \mathbf{r})$.

The single-molecule partition function of species i is given by

$$\mathcal{Q}_i = \frac{1}{V}\int d\mathbf{r} \; q_i(t,\mathbf{r}) e^{\omega_{\alpha_i(t)}(\mathbf{r})} q_i^\dagger(t,\mathbf{r}) \tag{S8}$$

for any t.

To determine phase coexistence, it is convenient to work in the semigrand canonical ensemble, where the thermodynamic control parameters are the chemical potentials of the various molecular species μ_i. The semigrand potential density $\mathcal{G}$ takes the form:[2,3]

$$\begin{aligned}\frac{N_1\mathcal{G}}{\rho_0 V k_\mathrm{B}T} = &-\sum_i e^{\mu_i/k_\mathrm{B}T} Q_i - \frac{1}{V}\int d\mathbf{r} \; \Bigg[\sum_\alpha N_1\omega_\alpha(\mathbf{r})\phi_\alpha(\mathbf{r}) \\ &-\frac{1}{2}\sum_{\alpha,\beta(\neq\alpha)} \chi_{\alpha\beta}N_1\phi_\alpha(\mathbf{r})\phi_\beta(\mathbf{r}) + \eta(\mathbf{r})N_1\left(1-\sum_\alpha \phi_\alpha(\mathbf{r})\right)\Bigg].\end{aligned} \tag{S9}$$

Similar to the average concentrations, only three of the four chemical potentials are indepen-

dent due to the incompressibility constraint. Thus, we set $\mu_1 = 0$. Extremizing Equation S9, we obtain the same set of SCFT equations as in Equation S2, except that the equations calculating the density fields are modified as follows:

$$\phi_\alpha(\mathbf{r}) = \sum_i \frac{e^{\mu_i/k_\mathrm{B}T}}{N_i} \sum_{t=1}^{N_i} \delta_{\alpha_i(t),\alpha}\, q_i(t,\mathbf{r}) e^{\omega_{\alpha_i(t)}(\mathbf{r})} q_i^\dagger(t,\mathbf{r}) \tag{S10}$$

After solving the SCFT equations, the average concentrations are given by $\bar{\phi}_i = e^{\mu_i/k_\mathrm{B}T} Q_i$.

To construct equilibrium phase diagrams, we numerically solve Eqs. S3-S5 and S10 for a set of candidate phases and compare their semigrand potentials. During this process, we also optimize the dimensions of the computational box to determine the optimal spatial periodicity for each phase. This optimization is performed concurrently with the field variables, using gradient descent followed by variable-cell Anderson mixing to accelerate convergence.[4,5] Unless otherwise specified, we include lamellae (LAM), double gyroid (DG), hexagonally packed cylinders (HEX), body-centered cubic (BCC) spheres, as well as the disordered (DIS) phase, as the default set of candidate phases in our calculations.

Random-Phase Approximation (RPA)

When studying the ordering of a system from the disordered state to an ordered state, it is informative to analyze the point at which the disordered state loses its linear stability, i.e., the spinodal point. This can be identified using the RPA.[6–9] The main task of RPA is to evaluate the inverse collective structure factor, $\mathbb{S}^{-1}(\mathbf{k})$, which corresponds to the second-order coefficient matrix of the expansion of the free-energy density (Equation S2) in terms of the composition fluctuations, $\delta\phi_\alpha(\mathbf{k})$, with wavevector k:

$$\frac{\mathcal{F}[\phi] - \mathcal{F}_\mathrm{homo}}{k_\mathrm{B}T\rho_0 V} = \frac{1}{2}\sum_{\mathbf{k}}\sum_{\alpha,\beta} \delta\phi_\alpha(\mathbf{k})\mathbb{S}^{-1}_{\alpha\beta}(\mathbf{k})\delta\phi_\beta(-\mathbf{k}) + \mathcal{O}\left(\delta\phi_\alpha^3\right), \tag{S11}$$

where $\mathcal{F}_{\text{homo}}$ represent the free energy for the homogeneous phase. For the quaternary system with four chemically distinct components, i.e., A, B, S and C, there are three independent composition fluctuations due to the incompressibility condition. Following the procedure of RPA for polymer mixtures,[9] the inverse collective structure factor matrix is written as

$$\mathbb{S}_{\alpha\beta}^{-1} = \begin{pmatrix} a_{\text{AA}} & a_{\text{AB}} & a_{\text{AS}} \\ a_{\text{AB}} & a_{\text{BB}} & a_{\text{BS}} \\ a_{\text{AS}} & a_{\text{BS}} & a_{\text{SS}} \end{pmatrix}, \tag{S12}$$

with matrix elements

$$\begin{aligned}
a_{\text{AA}} &= \frac{N_1\bar{\phi}_1 g_{\text{B1B1}}(k)}{D} + \frac{N_2\bar{\phi}_2 g_{\text{B2B2}}(k)}{D} + \frac{1}{N_{\text{C}}\bar{\phi}_{\text{C}} g_{\text{CC}}(k)} - 2\chi_{\text{AC}} && \text{(S13)}\\
a_{\text{AB}} &= \frac{-N_1\bar{\phi}_1 g_{\text{A1B1}}(k) - N_2\bar{\phi}_2 g_{\text{A2B2}}(k)}{D} + \frac{1}{N_{\text{C}}\bar{\phi}_{\text{C}} g_{\text{CC}}(k)} \\
&\quad -(-\chi_{\text{AB}} + \chi_{\text{AC}} + \chi_{\text{BC}}) && \text{(S14)}\\
a_{\text{AS}} &= \frac{1}{N_{\text{C}}\bar{\phi}_{\text{C}} g_{\text{CC}}(k)} - (\chi_{\text{AC}} - \chi_{\text{AS}} + \chi_{\text{SC}}), && \text{(S15)}\\
a_{\text{BB}} &= \frac{N_1\bar{\phi}_1 g_{\text{A1A1}}(k)}{D} + \frac{N_2\bar{\phi}_2 g_{\text{A2A2}}(k)}{D} + \frac{1}{N_{\text{C}}\bar{\phi}_{\text{C}} g_{\text{CC}}(k)} - 2\chi_{\text{BC}}, && \text{(S16)}\\
a_{\text{BS}} &= \frac{1}{N_{\text{C}}\bar{\phi}_{\text{C}} g_{\text{CC}}(k)} - (\chi_{\text{BC}} - \chi_{\text{BS}} + \chi_{\text{SC}}), && \text{(S17)}\\
a_{\text{SS}} &= \frac{1}{N_{\text{S}}\bar{\phi}_{\text{S}} g_{\text{SS}}(k)} + \frac{1}{N_{\text{C}}\bar{\phi}_{\text{C}} g_{\text{CC}}(k)} - 2\chi_{\text{SC}}, && \text{(S18)}
\end{aligned}$$

with

$$\begin{aligned}
D = {} & N_1^2\bar{\phi}_1^2\left[g_{\text{A1A1}}(k)g_{\text{B1B1}}(k) - g_{\text{A1B1}}(k)^2\right] \\
& + N_1N_2\bar{\phi}_1\bar{\phi}_2\left[g_{\text{A1A1}}(k)g_{\text{B2B2}}(k) - 2g_{\text{A1B1}}(k)g_{\text{A2B2}}(k) + g_{\text{A2A2}}(k)g_{\text{B1B1}}(k)\right] \qquad \text{(S19)} \\
& + N_2^2\bar{\phi}_2^2\left[g_{\text{A2A2}}(k)g_{\text{B2B2}}(k) - g_{\text{A2B2}}(k)^2\right].
\end{aligned}$$

Since we consider fluctuations around the spatially homogeneous, disordered phase, the functions $g_{\alpha\beta}$ depend only on the magnitude of the wavevector (k), and are related to correlation functions of ideal, noninteracting discrete Gaussian chains, given by:

$$g_{\alpha\alpha}(k) = \frac{2p(k)[p^{N_\alpha}(k) - 1] - N_\alpha p^2(k) + N_\alpha}{N_i^2[p(k) - 1]^2} \tag{S20}$$

and

$$g_{\alpha\beta}(k) = \frac{p(k)\left[p^{N_\alpha}(k) - 1\right]\left[p^{N_\beta}(k) - 1\right]}{N_i^2[p(k) - 1]^2}, \tag{S21}$$

Here, N_i denotes the total length of the polymer chain containing the blocks described by the correlation function, and $p(k)$ is the Fourier transform of the bond transition probability, Equation S7, $p(k) = \exp\left(-\frac{k^2 b^2}{6}\right)$.

The stability limit of the homogeneous phase is determined by the condition that the smallest eigenvalue of $\mathbb{S}^{-1}$ equals zero at a wavevector k^*. A nonzero k^* indicates an instability toward microphase separation at a finite length scale, whereas $k^* = 0$ corresponds to macrophase separation. The eigenvalues of the matrix in Equation S12 exhibit oscillatory behavior near $k = 0$. This oscillation complicates the accurate identification of the transition point at which k^* shifts from nonzero to zero. To resolve this issue, we introduce a cutoff $k^* R_e \leq 0.05$, treating all values below this threshold as indicative of macrophase separation.

Particle-Based Monte-Carlo Simulation

To study the dynamical and nonequilibrium behavior of the polymer blends, we perform Monte-Carlo (MC) simulations using the single-chain-in-mean-field (SCMF) algorithms. SCMF simulations approximately decouple soft, nonbonded interactions through quasi-instantaneous fields, dramatically improving computational efficiency and enabling the simulation of very

large system sizes.[10] In our simulations, we use the same molecular model as that used in the SCFT and RPA, i.e., the different molecules are modeled as chains composed of soft, highly coarse-grained segments connected by harmonic bonds. The usage of such a soft coarse-grained model in the simulations is very efficient in describing polymeric systems with a realistic value of invariant degree of polymerization,[11] $\sqrt{\bar{\mathcal{N}}} \equiv \rho_0 R_e^3/N_1$. Specifically, a large value of $\sqrt{\bar{\mathcal{N}}} = 380$ is used in all simulations in this work. The most complex system explored in our simulations is a quinary blend comprising two distinct AB diblock copolymers (A_1B_1 and A_2B_2), a volatile solvent (S), a nonvolatile solvent (C), and either a gas (G) or a nonsolvent (N). Each gas or nonsolvent molecule has the same number of beads (8) as each solvent molecule. We follow the same notational conventions used in SCFT to represent the various variables, with an additional set of parameters introduced for the fifth molecular species. These parameters are subscripted with the corresponding letter representing their molecular type (G or N).

A configuration of the soft, coarse-grained particle-based model is specified by the bead positions, $\mathbf{r_{it}}$, where the indices, i and t, run over all molecules and segments within a molecule, respectively. For each configuration, we define a set of concentration fields $\phi_\alpha(c)$ on a three-dimensional (3D) collocation grid with spacing $\Delta L = R_e/10$:[10,12]

$$\hat{\phi}_\alpha(\mathbf{c}) = \frac{1}{\rho_0 \Delta L^3} \sum_{it} \delta_{\alpha_i(t),\alpha}\, \Pi_{\mathbf{c}}(\mathbf{r}_{it}) \; . \tag{S22}$$

Here, the sum runs over all segments of type α and $\Pi_{\mathbf{c}}$ is the characteristic function of the grid cell, $\mathbf{c}$, i.e., it takes the value 1 if its argument $\mathbf{r}$ is within the cell $\mathbf{c}$, and 0 otherwise.

With the concentrations fields evaluated from the particle coordinates, we define the pairwise, nonbonded interactions:[10,12]

$$\frac{\mathcal{H}_{\text{nb}}}{\sqrt{\bar{\mathcal{N}}} k_{\text{B}} T} = \frac{\Delta L^3}{R_e^3} \sum_{\mathbf{c}} \left\{ \sum_{\alpha,\beta(\neq\alpha)} \frac{\chi_{\alpha\beta} N_1}{2} \hat{\phi}_\alpha(\mathbf{c}) \hat{\phi}_\beta(\mathbf{c}) + \frac{\kappa N_1}{2} \left[\sum_\alpha \hat{\phi}_\alpha(\mathbf{c}) - 1 \right]^2 \right\} , \tag{S23}$$

where the first term represents the pairwise interactions between unlike bead types, quantified by $\chi_{\alpha\beta}$, whereas the second term penalizes deviations of the total concentration from unity. In all simulations, we fix the parameter $\kappa N_1 = 85$, which is proportional to the inverse, isothermal compressibility.

All simulations are performed using the open-source software SOft coarse grained MC Acceleration (SOMA).[12] Using the strong bonded forces, we bias the local MC displacements of the beads,[13] resulting in Rouse-like dynamics.[14] The time unit, τ_R, is set by the time required for a linear, noninteracting polymer with N_1 segments to diffuse its end-to-end distance. For $N_1 = 64$, we measure $\tau_R = 34\,050$ Monte-Carlo steps (MCS).

To reduce computational cost, a quasi-two-dimensional (2D) simulation cell with dimensions $24 \times 21 \times 1 R_e^3$ is used for all simulations without solvents. For these simulations, periodic boundary conditions are applied in all directions. For systems with solvents, such as those involving EISA and NIPS, an extended third (z) dimension of $50R_e$ is employed, resulting in over half a billion ($\sim 6 \times 10^8$) soft coarse-grained particles per simulation. Leveraging the efficient parallelization capabilities of SOMA, each large-scale self-assembly by nonsolvent-induced phase separation (SNIPS) simulation, spanning a duration of $30\tau_R$, can be completed in approximately 4 days, using 8 NVIDIA A100 GPUs. In the SNIPS simulations, periodic boundary conditions are applied along the lateral x and y dimensions, while two impenetrable walls are placed at the top and bottom along the z dimension of the simulation cell. Solvent evaporation during EISA is modeled by defining a conversion zone within the simulation cell.[15] At the beginning of the simulation, the conversion zone is a thin layer at the top of the simulation cell, and it dynamically follows the surface of the polymer solution to maintain a distance of $0.6R_e$ away from the polymer-gas interface. If the center of mass of a volatile solvent molecule enters this zone, it is converted into a gas molecule.[15] During NIPS, the movement of the conversion zone stops, and a similar mechanism is employed to convert solvent molecules into nonsolvent molecules.[16]

When simulating EISA or NIPS, the vitrification of polymer chains plays a crucial role

in capturing the formation of frozen, nonequilibrium structures at high polymer concentrations.[17] To account for this effect in simulations, a concentration-dependent mobility modifier is applied to the polymer segments:[16,18,19]

$$m(\{\phi_\alpha\}) = \frac{1}{2}\left[1 + \tanh\left(\frac{1 - \sum_\alpha a_\alpha \phi_\alpha}{3}\right)\right], \tag{S24}$$

where the coefficients $a_\alpha = [17, 17, -40, -40, 0, 0]$ are selected for $\alpha = \{\mathrm{A, B, S, C, G, N}\}$ to ensure that the polymer mobility drops sharply once the local polymer concentration exceeds a threshold.

In addition to the mobility modifier, the MC update frequency for segments of different molecular species can be tuned individually, independent of the local copolymer concentration, to adjust their dynamical asymmetry.[19]

Additional Figures

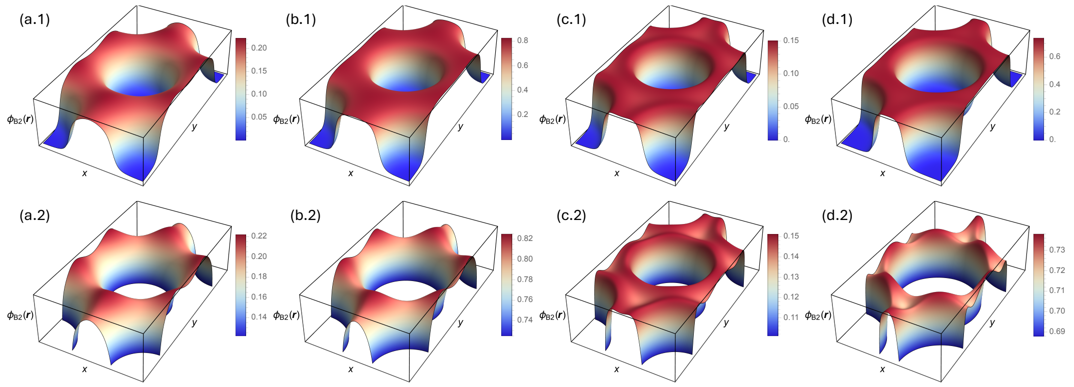

Figure S1: 3D visualizations of the B_2-block density in HEX phases formed in binary A_1B_1/A_2B_2 blends at $f_1 = 0.3125$, $\gamma_2 = 1.25$, and $\chi_{AB}N_1 = 30$, for various values of f_2 and $\bar{\phi}_2$. The third dimension, representing the density magnitude, is combined with a color scale to enhance visualization of spatial density variations. Each row shows a pair of subfigures, where the bottom panel (x.2) offers a zoomed-in view along the third dimension of the top panel (x.1) to highlight fine details in high-density regions. Subfigures (a–b) correspond to $f_2 = 0.3125$, and (c–d) to $f_2 = 0.5$, with $\bar{\phi}_2 = 0.2$ for (a, c) and $\bar{\phi}_2 = 0.8$ for (b, d).

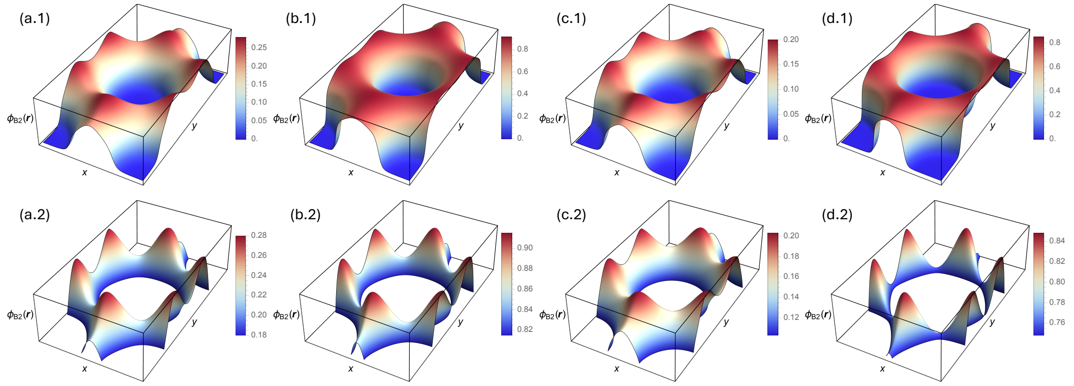

Figure S2: 3D visualizations of the B_2-block density in HEX phases formed in binary A_1B_1/A_2B_2 blends, using the same parameters as in Figure S1, except with $\gamma_2 = 2.25$.

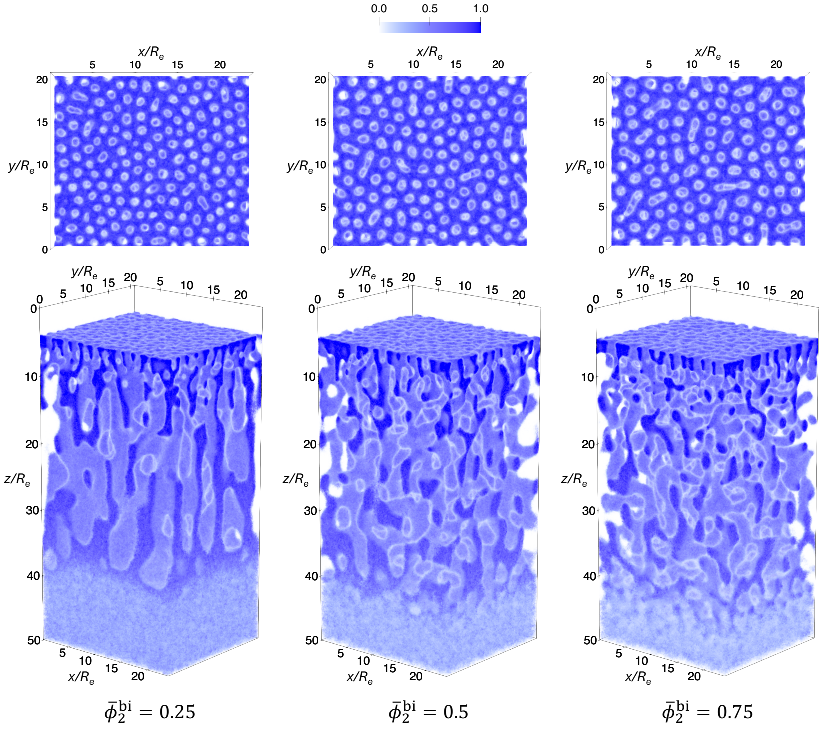

Figure S3: Normalized 3D density profiles at the end of NIPS simulations with $\gamma_2 = 2.25$ and optimized parameters at different $\bar{\phi}_2^{\mathrm{bi}}$ values. The $\bar{\phi}_{\mathrm{P}}^{(0)}$ values are determined using method 2 described in the main text. The top panel shows a top-down view of the corresponding morphology in the bottom panel. The morphologies at $\bar{\phi}_2^{\mathrm{bi}} = 0$ and 1 are identical to those in Figure 20 of the main text, as the two methods for determining $\bar{\phi}_{\mathrm{P}}^{(0)}$ yield the same values at these $\bar{\phi}_2^{\mathrm{bi}}$ points, corresponding to identical simulations.

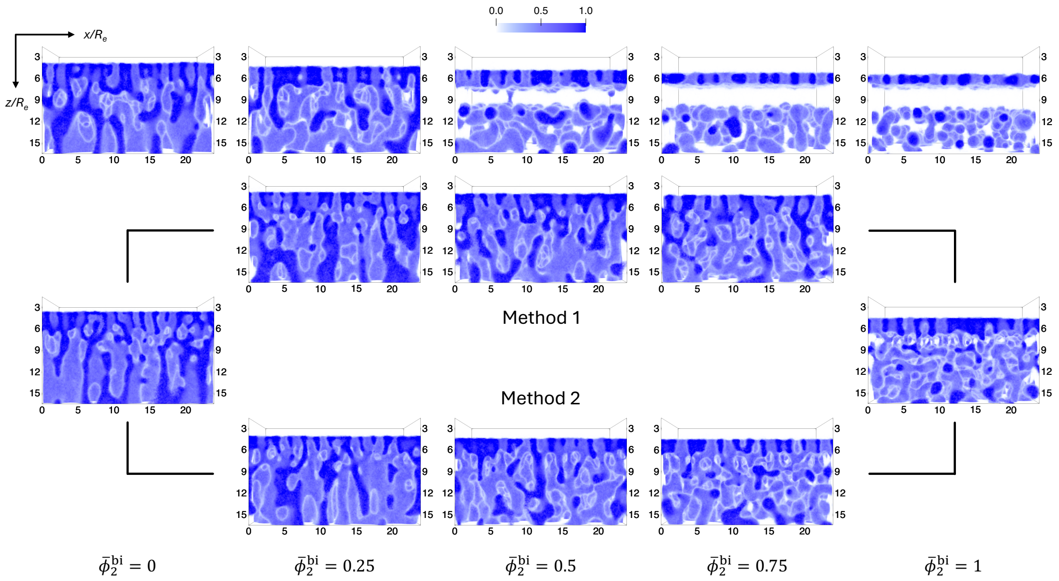

Figure S4: Enlarged snapshots from Figure 18 (top panel), Figure 20 (bottom panel, top branch) and Figure S3 (bottom panel, bottom branch), highlighting the connection zones between the top layer and substructure of the membrane.

Processing of Simulation Data

Post-Processing of Density Data from Simulations

In contrast to the smooth 2D density profiles predicted by SCFT, those obtained from SCMF simulations exhibit noticeable thermal fluctuations, in the form of random transient A-rich patches with x-y areas ranging up to $0.2R_e^2$. To avoid misidentifying these fluctuations as genuine A cylinders, we post-process each 2D density snapshot using a three-step procedure: First, we binarize the 2D density profile using the threshold condition $\phi_{\mathrm{A}}(\mathbf{r}) > \phi_{\mathrm{B}}(\mathbf{r})$. Second, we denoise the 2D binary profile by applying an erosion operation to remove boundary pixels from A-rich domains, thereby eliminating most small, spurious patches. Third, we perform a dilation step to restore the area lost from genuine A cylinders during erosion. The resulting density profile is then used to determine the individual cylinder radii and their average. In calculating the average radius, highly distorted domains are excluded. To quantify domain shape, we first identify its geometric center, and classify a domain as highly distorted if the ratio of the longest to shortest distance from the center to any boundary point exceeds 3. In Figure S5, we present 2D densities after different post-processing steps for two simulations (quench and EISA) at $t = 12\tau_R$, with parameters described in the caption.

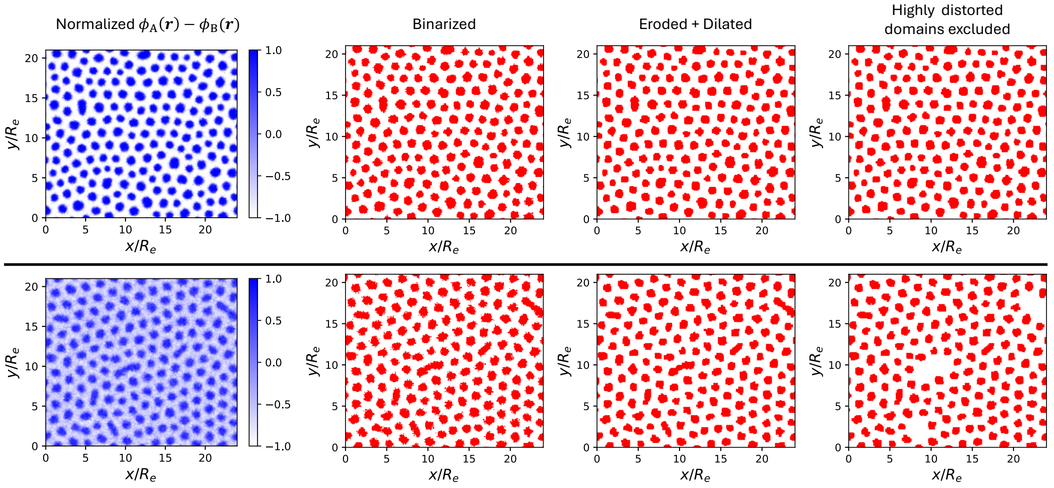

Figure S5: 2D densities after different post-processing steps for two simulations at $t = 12\tau_R$: quench (top panel) and EISA (bottom panel). For both simulations, $f_1 = f_2 = 0.3125$, $\gamma_2 = 1.5$ and $\bar{\phi}_2^{\text{bi}} = 0.5$. After binarization, the A-rich regions are colored in red. For the selected quench simulation, no domain is classified as highly distorted, resulting in identical binarized profiles in the third and fourth columns. For the EISA simulation, the slice is taken from the z index of 48, at which the maximum laterally averaged cylinder radius is identified.

Extraction of Average Cylinder Radii from EISA Simulations

The radii of the cylinders formed in the top layer during EISA vary along the z direction. For each fixed z position, an average cylinder radius can be evaluated. As examples, Figure S6 presents the average radii obtained from 2D x-y slices at various z positions for systems with $\bar{\phi}_2^{\mathrm{bi}} = 0$ and 0.5 (with $\gamma_2 = 1.5$). Generally, we observe that the average radius initially increases with z, reaches a maximum, and then decreases. To determine a representative average radius for the entire EISA top layer, we average the laterally averaged radii over a selected z range centered around the maximum. For each system, this z range is chosen such that the relative standard deviation (RSD) of the laterally averaged radii within the range is just below 5%.

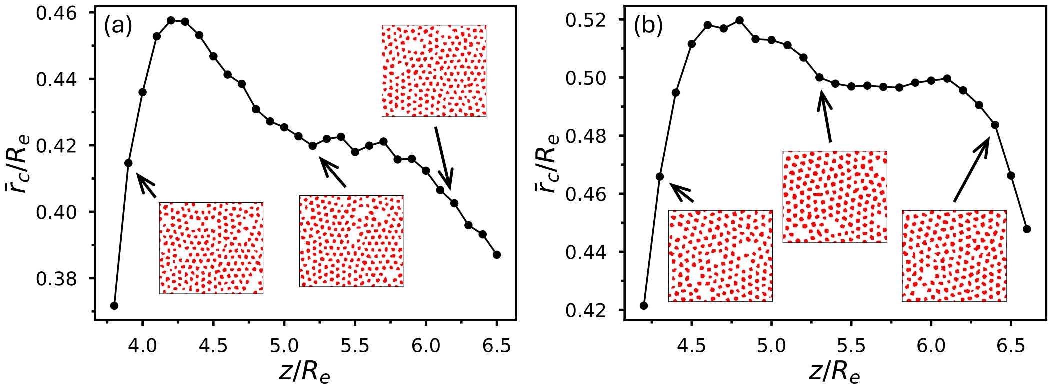

Figure S6: Average radii measured in x–y cross sections as a function of z within the top layer at the end of the EISA simulations with $\gamma_2 = 1.5$ and $\bar{\phi}_2^{\mathrm{bi}}$ =(a) 0 and (b) 0.5. The z range around the maximum is selected such that the RSD of the laterally averaged radii within this range remains just below 5%. The top-layer average radius is then calculated by averaging the laterally averaged radii over this selected range. Binarized maps based on post-processed simulation density data are shown at representative z positions, where the A-rich regions are colored in red.

References

(1) Fredrickson, G. *The equilibrium theory of inhomogeneous polymers*; Oxford University Press, 2006.

(2) Shi, A.-C. *Variational Methods in Molecular Modeling*; Springer, 2016; pp 155–180.

(3) Xie, J.; Shi, A.-C. Theory of complex spherical packing phases in diblock copolymer/homopolymer blends. *Macromolecules* **2023**, *56*, 10296–10312.

(4) Thompson, R. B.; Rasmussen, K. O.; Lookman, T. Improved convergence in block copolymer self-consistent field theory by Anderson mixing. *The Journal of chemical physics* **2004**, *120*, 31–34.

(5) Arora, A.; Morse, D. C.; Bates, F. S.; Dorfman, K. D. Accelerating self-consistent field theory of block polymers in a variable unit cell. *The Journal of chemical physics* **2017**, *146*.

(6) Leibler, L. Theory of microphase separation in block copolymers. *Macromolecules* **1980**, *13*, 1602–1617.

(7) Hong, K. M.; Noolandi, J. Theory of phase equilibriums in systems containing block copolymers. *Macromolecules* **1983**, *16*, 1083–1093.

(8) Whitmore, M. D.; Noolandi, J. Theory of phase equilibria in block copolymer-homopolymer blends. *Macromolecules* **1985**, *18*, 2486–2497.

(9) Xie, J.; Shi, A.-C. Phase behavior of triblock copolymer and homopolymer blends: Effect of copolymer topology. *Physical Review Materials* **2024**, *8*, 015601.

(10) Daoulas, K. C.; Müller, M. Single chain in mean field simulations: Quasi-instantaneous field approximation and quantitative comparison with Monte Carlo simulations. *The Journal of chemical physics* **2006**, *125*.

(11) Müller, M. Studying amphiphilic self-assembly with soft coarse-grained models. *Journal of Statistical Physics* **2011**, *145*, 967–1016.

(12) Schneider, L.; Müller, M. Multi-architecture Monte-Carlo (MC) simulation of soft coarse-grained polymeric materials: SOft coarse grained Monte-Carlo Acceleration (SOMA). *Computer Physics Communications* **2019**, *235*, 463–476.

(13) Müller, M.; Daoulas, K. C. Single-chain dynamics in a homogeneous melt and a lamellar microphase: A comparison between Smart Monte Carlo dynamics, slithering-snake dynamics, and slip-link dynamics. *The Journal of chemical physics* **2008**, *129*.

(14) Rouse Jr, P. E. A theory of the linear viscoelastic properties of dilute solutions of coiling polymers. *The Journal of Chemical Physics* **1953**, *21*, 1272–1280.

(15) Dreyer, O.; Ibbeken, G.; Schneider, L.; Blagojevic, N.; Radjabian, M.; Abetz, V.; Müller, M. Simulation of Solvent Evaporation from a Diblock Copolymer Film: Orientation of the Cylindrical Mesophase. *Macromolecules* **2022**, *55*, 7564–7582, DOI: 10.1021/acs.macromol.2c00612.

(16) Blagojevic, N.; Müller, M. Simulation of membrane fabrication via solvent evaporation and nonsolvent-induced phase separation. *ACS Applied Materials & Interfaces* **2023**, *15*, 57913–57927.

(17) Müller, M.; Abetz, V. Nonequilibrium processes in polymer membrane formation: Theory and experiment. *Chemical reviews* **2021**, *121*, 14189–14231.

(18) Müller, M.; Smith, G. D. Phase separation in binary mixtures containing polymers: A quantitative comparison of single-chain-in-mean-field simulations and computer simulations of the corresponding multichain systems. *J. Polym. Sci., Part B: Polym. Phys.* **2005**, *43*, 934–958, DOI: 10.1002/polb.20385.

(19) Blagojevic, N.; Das, S.; Xie, J.; Dreyer, O.; Radjabian, M.; Held, M.; Abetz, V.; Müller, M. Toward Predicting the Formation of Integral-Asymmetric, Isoporous Diblock Copolymer Membranes. *Advanced Materials* **2024**, *36*, 2404560.
